# Combining Density Functional Embedding Theory and DMRG-NEVPT2 to Treat Large Active Spaces: Addressing Electronic Structure Complexity in Single-Atom Alloys

**DOI:** 10.1021/acs.jctc.5c02119

**Published:** 2026-02-19

**Authors:** Phillips Hutchison, Ziyang Wei, Emily A. Carter

**Affiliations:** † Department of Mechanical and Aerospace Engineering, 6740 Princeton University, 41 Olden Street, Princeton, New Jersey 08544, United States; ‡ Andlinger Center for Energy and the Environment and Program in Applied and Computational Mathematics, Princeton University, Princeton, New Jersey 08544-5263, United States

## Abstract

Single-atom alloys (SAAs) are an increasingly popular platform for heterogeneous catalysis because of their distinct electronic structures and ability to break catalytic linear scaling relationships. This popularity has led to a proliferation of computational studies probing SAA reactivity at the density functional theory (DFT) level. However, some phenomena such as photo- and electrocatalysis require use of electronic structure methods beyond DFT; such studies are both rare and fundamentally challenging. Density functional embedding theory (DFET)/embedded correlated wavefunction (ECW) studies of reactions on metal surfaces have been shown to provide a reliable way to correct for DFT-related errors. DFET/ECW studies of chemistry involving SAAs, however, could require active spaces beyond the capabilities of traditional multireference methods when transition-metal dopants give rise to many degenerate states. To overcome this limitation, we combined our DFET/ECW methodology with the density matrix renormalization group (DMRG) complete active space self-consistent field (DMRGSCF) and DMRG *N*-electron valence state second-order perturbation theory (DMRG-NEVPT2) methods in the PySCF code. Using embedded DMRGSCF and embedded DMRG-NEVPT2, we analyze CO adsorption on Ni-, Rh-, Pd-, and Pt-doped Ag(100) with different active spaces. We show that the active spaces approachable with conventional multireference methods lead to overbinding of CO due to an inability to treat all of the dopant d-orbitals on equal footing. Larger active spaces, which are easily treated by both DMRGSCF and DMRG-NEVPT2, yield much more reasonable adsorption free energies. Our findings suggest that future multireference calculations of these systems should similarly employ active spaces containing all of the dopant d-orbitals along with sp-band orbitals of the host metal near the Fermi level. Emb-DMRG-NEVPT2 is a method that can be broadly applied to study catalytic reactions on metal surfaces when large active spaces are required.

## Graphical abstract

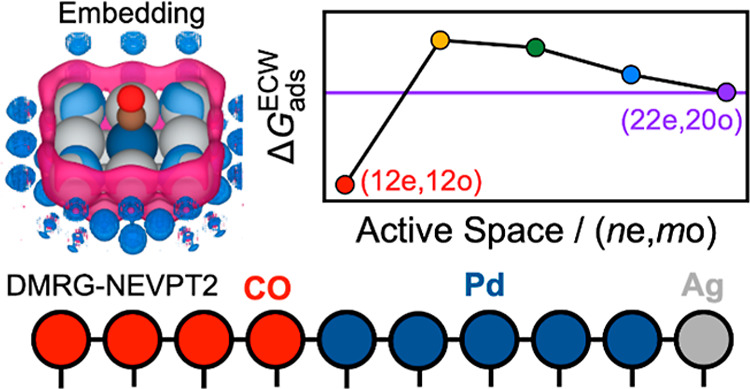

## Introduction

In the search to identify heterogeneous catalysts that are more active and selective than traditional catalysts, many efforts have been directed toward developing single-atom catalyst systems.^1–3^ The single-atom alloy (SAA) framework, where a reactive dopant metal species is atomically dispersed within a more chemically inert host metal, is one such example of this class of catalysts.^1,4–8^ SAAs have been shown to be active and selective for desirable dehydrogenation reactions such as ethane dehydrogenation and dry ethanol dehydrogenation.^5,8–10^ Moreover, the SAA framework has gained great popularity in the past few years due to the well-documented ability of SAAs to break traditional linear scaling relationships.^6,11^ This ability to break linear scaling relationships is often attributed to the different adsorption characteristics of the dopants and host metals. While the dopant can be effective for binding and dissociating reactant chemicals, the host may present more facile desorption for subsequent reactive intermediates, yielding enhanced catalytic properties as compared with pure surfaces of either metal.

Beyond their desirable catalytic behavior, SAAs are noted for having unique electronic structures that are distinct from those of traditional alloys. Previous experimental investigations have suggested that in SAA materials, poor mixing of the dopant and host metal orbitals can give rise to sharp, narrow features in X-ray photoemission spectra (XPS) that correspond to highly degenerate dopant metal d-states.^12,13^ Such a phenomenon, sometimes referred to as “band narrowing,” also has been seen in density functional theory (DFT) investigations of SAAs.^6,14^ Together, these insights have prompted researchers to view these dopant atoms as behaving like isolated atoms and to rationalize their chemistries through this lens. However, most studies of SAAs to date are based on generalized gradient approximation DFT studies,^9,11,15–18^ which have well-documented failings for metallic systems, such as leading to overly bound adsorbates.^19,20^

A more accurate treatment of chemistry on metallic and SAA surfaces requires accounting for dynamic electron correlation, which can be accomplished through many-body electronic structure theories. While more accurate, higher computational cost typically limits many-body approaches to smaller atomic-scale systems than those typically used to model extended metal surfaces. Approaches based on embedding, such as density functional embedding theory (DFET)^21–23^ and embedded correlated wavefunctions^24^ (ECW) are designed to address this challenge. DFET and ECW approaches have been shown to be successful in balancing a treatment of the extended metallic environment with the treatment of dynamic correlation.^25–36^ Previous work focusing on SAA systems as models for antenna-reactor plasmonic nanoparticles has shown that the predictions of multireference wavefunction approaches can differ significantly from the predictions of DFT calculations.^32,37,38^

A multireference wavefunction approach, such as the complete active space self-consistent field (CASSCF) method,^39^ overcomes the limitations of Hartree–Fock theory when applied to metallic systems, such as divergence at the Fermi level^40^ and the spurious appearance of charge-density and spin-density waves.^41^ A prominent limitation in CASSCF studies is the unfavorable scaling with system size due to exactly solving the full configuration-interaction (FCI) expansion within the active space. While multireference methods allow for systematic improvement of the wavefunction by including relevant orbitals in the active space, the largest computationally tractable active spaces typically have sizes of roughly 16 electrons in 16 orbitals, also written as (16e,16o). In addition to the static correlation treated by CASSCF, quantitative accuracy in metallic clusters requires dynamic correlation corrections from (at least) multireference second-order perturbation theory (MRPT2), such as *N*-electron valence state second-order perturbation theory (NEVPT2).^42^ These corrections typically are limited to even smaller active spaces than CASSCF. With CO on a Pd_1_Ag_12_ cluster, for example, NEVPT2 calculations based on a CASSCF reference wavefunction are not tractable with active spaces larger than (13e,13o). Further, prior ECW studies of metallic clusters have shown that large active space methods (e.g., adaptive sampling CI) without MRPT2 corrections do not recover enough dynamical correlation for quantitative accuracy.^26^ Transition-metal SAAs present a unique challenge to traditional multireference methods. Given the experimental XPS spectra^12,13^ and previous theoretical insights,^6^ it is likely that all the dopant d-orbitals will be important to a high-fidelity description of chemistry on SAAs. As a result, even simple reactions such as CO adsorption on the dopant metal could require active spaces on the order of (20e,18o), which would not be possible to treat with traditional NEVPT2 based off a CASSCF reference wavefunction.

In the past three decades, there have been impressive developments in multireference quantum chemistry based on the density matrix renormalization group (DMRG).^43–51^ CASSCF methods where DMRG is used to approximately solve the FCI expansion (DMRGSCF) are even capable of treating active spaces with up to 100 orbitals.^49,52^ DMRG approaches are also appealing, given their variational nature and given that only a single parameter, the maximum bond dimension *M*, controls the convergence. These features also allow DMRGSCF energies to be reliably extrapolated to infinite bond dimension, recovering the exact solution.^53,54^ Especially pertinent to multireference studies of metallic systems, DMRG-based methods have many established extensions to MRPT2 calculations.^55–58^ Given these considerations, it is clear that DMRG-based multireference methods present a blend of accuracy and flexibility that will make them applicable to metallic systems requiring large active spaces. In this work, we combine DFET/ECW methods with DMRGSCF and DMRG strongly contracted NEVPT2 (DMRG-sc-NEVPT2) as implemented in PySCF^59,60^ and Block2.^61^ We use these methods to study CO adsorption on Nickel (Ni), Rhodium (Rh), Palladium (Pd), and Platinum (Pt) doped Silver (Ag) SAAs by systematically expanding our active spaces. Our studies show that with these SAAs, incorporating all the dopant metal d-orbitals plus a pair of host metal orbitals into the active space (to describe the Fermi level states properly)^26^ is needed for quantitative accuracy in multireference studies. This work has important implications for active space selection in future multireference studies of SAAs and describes a reliable approach for generating large active spaces in metallic systems.

## Theoretical Background

### Density Matrix Renormalization Group Complete Active Space Self-Consistent Field

As mentioned above, in DMRGSCF, the exact solution to the FCI expansion within a set of active orbitals is replaced by an approximate solution afforded by the DMRG sweep algorithm. We will briefly review the basics of the DMRG sweep algorithm as it pertains to quantum chemistry, but more comprehensive literature on the theoretical underpinnings of this method can be found in the reviews of Sharma and Chan,^48,62^ Schöllwock,^47,63^ and Reiher and co-workers.^46,50,51^ To understand the DMRG procedure, we first write a general FCI wavefunction within a set of *k* orbitals in its occupation number representation

1 $$|\Psi \rangle =\sum_{\sigma_{1}\sigma_{2}\sigma_{3}...\sigma_{k}}C_{\sigma_{1}\sigma_{2}\sigma_{3}...\sigma_{k}}|\sigma_{1}\sigma_{2}\sigma_{3}...\sigma_{k}\rangle$$

where σ is the physical dimension of the system, the subscripts index the orbitals, $C_{\sigma_{1}\sigma_{2}\sigma_{3}...\sigma_{k}}$ are the FCI expansion coefficients, |σ_1_σ_2_σ_3_...σ_*k*_⟩ is the occupation number vector, and the summation runs over all occupation number vectors. For quantum chemistry, the physical dimension is four with possible values of |0⟩, |α⟩, |β⟩, or |αβ⟩, which represent the allowed occupancies of each orbital. We may recast this wavefunction into a matrix product state (MPS) as

2 $$|\Psi_{MPS}\rangle =\sum_{\sigma_{1}\sigma_{2}\sigma_{3}...\sigma_{k}}\sum_{a_{1}...a_{k-1}}A_{{1a}_{1}}^{\sigma_{1}}A_{a_{1}a_{2}}^{\sigma_{2}}A_{a_{2}a_{3}}^{\sigma_{3}}...A_{a_{k-1}1}^{\sigma_{k}}|\sigma_{1}\sigma_{2}\sigma_{3}...\sigma_{k}\rangle$$

where $A_{a_{i-1}a_{i}}^{\sigma_{i}}$ is a three-index tensor for the *i*-th orbital. In eq (2), we have introduced an auxiliary index *a* called the bond dimension along which we contract the matrices. The MPS corresponds to representing the orbitals in our quantum chemical problem as sites on a lattice with the auxiliary indices representing the entanglements between the orbitals. To keep the computation tractable, we limit the bond dimension to a maximum value *M* with larger values of *M* allowing us to incorporate more orbital entanglement in the optimized MPS. As such, each $A_{a_{i-1}a_{i}}^{\sigma_{i}}$ , except the first and the last, is a vector of four *M* × *M* matrices. To maintain the correct dimensionality, the first matrix consists of four row vectors of length *M* while the last matrix consists of four column vectors of length *M*. Each matrix is associated with one of the possible occupancies of the *i*-th orbital (i.e., |0⟩, |α⟩, |β⟩, or |αβ⟩). The DMRG sweep algorithm proceeds as a site-by-site optimization moving along the one-dimensional lattice. As one site tensor is optimized, the *M* states most important to the overall wavefunction (i.e., those with the highest weights in the reduced density matrix) are retained as the updated renormalized basis states. The subsequent sites are then optimized by the same procedure until the end of the chain is reached, at which point the direction of the sweep is reversed. This is repeated until convergence is reached. The cost and accuracy of the DMRG calculation are limited by the maximum allowed bond dimension *M* and at infinitely large *M* the exact solution is recovered, though in practice finite values of *M* give acceptable accuracy.

### Density Functional Embedding Theory and Embedded Correlated Wave Functions

Our calculations are performed within the DFET and ECW frameworks, where an embedding potential *V*_emb_ is applied to a region of interest, here a metal cluster. This embedding potential *V*_emb_ represents the physics of the interaction between the metal cluster and the extended metallic environment that would be present in a fully periodic slab (Figure 1) treated at the level of planewave (PW) DFT. *V*_emb_ is obtained by maximizing an extended Wu-Yang functional,^64^ *W*[*V*_emb_], with respect to variations in *V*_emb_.

3 $$W\left[V_{emb}\right]=E_{cluster}\left[\rho_{cluster},V_{emb}\right]+E_{env}\left[\rho_{env},V_{emb}\right]-\int V_{emb}\rho_{tot}dr$$

*E*_cluster_ is the energy of the region of interest denoted as the cluster, *E*_env_ is the energy of the (metallic) environment, ρ_cluster_ is the electron density of the cluster, ρ_env_ is the electron density of the metallic environment, and ρ_tot_ is the electron density of the reference system. At the maximum of this functional, the electron densities of the metal cluster and the environment, both in the presence of *V*_emb_, sum to the electron density of the fully periodic slab (i.e., ρ_tot_ = ρ_cluster_[*V*_emb_]+ρ_env_[*V*_emb_]). By extension, applying *V*_emb_ to the metal cluster recreates the same electron density distribution that would be present in the fully periodic slab calculation. *V*_emb_ modifies the one-electron Hamiltonian and allows for CW calculations on the metal cluster without fully sacrificing the effect of the metallic environment. Within this formalism, the ECW energy (*E* ^ECW^) is obtained as.

4 $$E^{ECW}=E_{slab}^{PW-DFT}+E_{cluster}^{emb-CW}-E_{cluster}^{emb-DFT}$$

where *E*_slab_^PW–DFT^ is the energy of the fully periodic slab treated at the PW-DFT level without empirical dispersion corrections and *E*_cluster_^emb–CW^ is the energy of the metal cluster in the presence of *V*_emb_ treated at a CW level of theory. In this paper, the correlated method will always be DMRG-sc-NEVPT2 (i.e., *E*_cluster_^emb–CW^ = *E*_cluster_^emb–DMRG‑sc‑NEVPT2^). Lastly, *E*_cluster_^emb–DFT^ is the energy of the metal cluster in the presence of *V*_emb_ and treated at the DFT level of theory without dispersion corrections. With the embedded DFT term, there is a choice between atom-centered Gaussian-type orbital (GTO) and PW basis sets. In light of this, we differentiate between ECW energies with emb-DFT terms from either PW or GTO basis sets as

5 $$E^{ECW,PW}=E_{slab}^{PW-DFT}+E_{cluster}^{emb-CW}-E_{cluster}^{emb-PW-DFT}$$

6 $$E^{ECW,GTO}=E_{slab}^{PW-DFT}+E_{cluster}^{emb-CW}-E_{cluster}^{emb-GTO-DFT}$$

In (5) and (6), the superscripts GTO and PW indicate the type of basis set used in the emb-DFT calculation. For our studies, we employ a PW basis set for the emb-DFT calculations involving Rh, Pd, and Pt dopants, and we use a GTO basis set for the Ni dopant. In general, we find that the PW basis set provides a better cancellation of error for Pd and Pt dopants. We use the GTO basis set in emb-DFT calculations for Ni because the Ni atom is a triplet at the emb-DMRGSCF level when CO is desorbed from the embedded cluster, and enforcing this spin for the embedded cluster with PW basis set results in spurious magnetization of the Ag host. Therefore, use of the GTO basis allows us to enforce the same spin state in the energy contributions from embedded calculations despite a nonmagnetic state being favored in the PW-DFT slab calculation. For completeness, we report results from the ECW calculations with a GTO basis set for the emb-DFT calculations involving Rh, Pd, and Pt and with a PW basis set for the emb-DFT calculations involving Ni in the Supporting Information. We then can compute CO adsorption free energies within an ECW framework as

7 $$\Delta G_{ads}^{ECW,PW}=E^{ECW,PW}\left({CO}_{ads}\right)-E^{ECW,PW}\left({CO}_{des}\right)+\Delta ZPE-T\Delta S-\int_{0}^{T}C_{P,CO}dT$$

8 $$\Delta G_{ads}^{ECW,GTO}=E^{ECW,GTO}\left({CO}_{ads}\right)-E^{ECW,GTO}\left({CO}_{des}\right)+\Delta ZPE-T\Delta S-\int_{0}^{300}C_{P,CO}dT$$

where Δ*G*_ads_^ECW^ is the ECW adsorption free energy, CO_ads_ corresponds to CO adsorbed on the SAA, CO_des_ corresponds to CO desorbed from the SAA (details below), ΔZPE is the change in zero-point energy (ZPE) between adsorbed and desorbed states, *T*Δ*S* are the corresponding entropic contributions (details below), and the last term accounts for the heat capacity of CO in the gas phase. The CO constant pressure heat capacity was obtained from the NIST chemistry webbook,^65^ with the correction amounting to 0.09 eV at *T* = 300 K. We neglect the change in the heat capacity of the metal substrate upon adsorption, as the change is expected to be negligible and therefore will cancel out. We extrapolate adsorption free energies for Pd, Rh, and Ni dopants to the complete basis set (CBS) limit using the method proposed by Helgaker.^66^ We fit our adsorption energies to a function of the form a + bX^–3^ where X is the cardinal number for correlation-consistent basis sets (e.g., two for aug-cc-pVDZ and three for aug-cc-pVTZ), b is a fitting parameter, and a is the adsorption free energy at the CBS limit.

**Figure 1:**
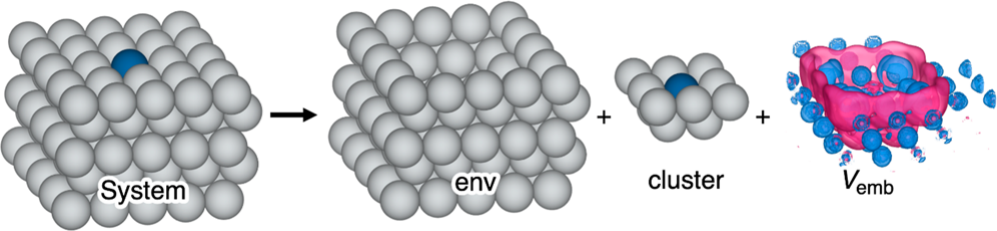
Schematic of the decomposition of a metallic slab into an environment (env), a cluster, and a unique embedding potential (*V*_emb_) describing the interactions between the cluster and the environment. Here, the system is a five-layer 5 × 5 supercell Ag(100) slab with a single Pd atom (blue) substituting a surface layer Ag atom (gray) and the cluster has a stoichiometry of Pd_1_Ag_12_. The optimized embedding potential is rendered as an isosurface with blue regions showing repulsive interactions (+0.23 V) and pink regions showing attractive interactions (−0.23 V).

## Computational Details

### Periodic DFT Calculations

Our models for Ni, Pd, and Pt SAAs were based on five-layer 5 × 5 supercell Ag(100) surface slabs with one dopant metal atom substituting a surface layer Ag atom. For the Rh-doped Ag(100) SAA, our primary model was a four-layer 5 × 5 slab with one Rh substituting a surface layer Ag. This slab dimension for the Rh dopant was chosen such that the overall slab would have an even number of electrons (to avoid any artificial spin-polarization in the host metal). In all slabs, the bottom two layers of Ag(100) were frozen in their equilibrium bulk positions, as determined within DFT-PBE with the numerical settings described below. To examine CO adsorption/desorption, we optimized geometries for the CO adsorbed on the dopant metal and geometries with CO desorbed from the dopant metal, separated by 12 Å (i.e., a supermolecule approach) with a total unit-cell length of 38 Å, which provides for ∼20 Å of vacuum between the periodic slab images. All optimized geometries were confirmed as minima by a frequency calculation that included the CO, the dopant metal, and the eight surface-layer Ag atoms surrounding the dopant (Figure 1). The calculated frequencies were used to evaluate the ZPE and entropic contributions to the Gibbs Free energy at 300 K. For adsorbed CO, we included only vibrational entropy contributions from CO and the nine atoms from the metal surface that would comprise the first layer of the embedded cluster. For desorbed CO, we used the experimental entropy obtained from the NIST chemistry webbook^65^ for the CO itself and only the vibrational entropy of the same nine atoms of the metal surface. All ZPE and thermal entropy corrections are given in Table S1 in the Supporting Information. All periodic DFT calculations were performed spin-polarized in VASP v6.4.3 ^67–69^ using the PBE functional^70^ within the projector augmented-wave (PAW) framework.^71–73^ The valence electron configurations solved for self-consistently in the presence of the PAW potentials were (2s^2^ 2p^2^) for C, (2s^2^ 2p^4^) for O, (3d^9^ 4s^1^) for Ni, (4d^8^ 5s^1^) for Rh, (4d^9^ 5s^1^) for Pd, (4d^10^ 5s^1^) for Ag, and (5d^9^ 6s^1^) for Pt. Geometry optimizations employed D3­(BJ)^74,75^ dispersion corrections and dipole corrections. Our PW basis had a 660 eV kinetic energy cutoff, and we employed 5 × 5 × 1 Γ-point-centered *k*-point sampling. We use Methfessel-Paxton smearing^76^ for the electronic states with a width of 0.09 eV. This level of theory will be referred to as PW-DFT+D3 below. All energies and forces were converged to a threshold of 1.0 × 10^–6^ and 0.01 eV/Å, respectively. The calculated lattice constant of Ag was 4.07 Å which agrees well with the experimentally measured value of 4.09 Å.^77^

### DFET/ECW Calculations

To generate our embedding potentials, we carved out 13-atom clusters from optimized slabs without adsorbates. The cluster consisted of the dopant metal atom, the eight Ag atoms surrounding the dopant in the surface layer, and the four Ag atoms contacting the dopant in the first subsurface layer (Figure 1). This size of the cluster was chosen so that the embedding potential would not contact the periodic boundary of the supercell. For the embedded calculations with the CO, we took the DFT-slab optimized positions of the CO and shifted them to the same position relative to the single-atom dopant on the embedded cluster (i.e., the static surface approximation).^35^ Embedded clusters treated at the PW-DFT level employed Γ-point-only *k*-point sampling. The embedding potential for Pd_1_Ag_12_ is shown in Figure 1 and the remaining embedding potentials are shown in Figures S1–S3 in the Supporting Information.

All emb-DMRGSCF, emb-DMRG-sc-NEVPT2, and emb-GTO-DFT calculations were performed in a locally modified version of PySCF v2.9.0 with an interface to Block2. GTO calculations were performed with both aug-cc-pVDZ (AVDZ) and aug-cc-pVTZ (AVTZ) basis sets for C, O, and Ni.^78^ For Rh, Pd, Ag, and Pt, we used aug-cc-pVDZ-PP and aug-cc-pVTZ-PP basis sets, representing the 28 core electrons of Rh, Pd, and Ag with ECP28MDF effective core potentials and the 60 core electrons of Pt with an ECP60MDF effective core potential, both of which are fully relativistic.^79^ All GTO basis sets were obtained from the Basis Set Exchange repository.^80,81^ Our embedding potentials were converted into one-electron integrals for a given GTO basis set using the EmbeddingIntegralGenerator code.^82^ All calculations employed density fitting with AVDZ calculations using def2-TZVPP-jkifit auxiliary basis sets and all AVTZ calculations using def2-QZVPP-jkfit auxiliary basis sets.^83^ All initial emb-DMRGSCF calculations were performed with a maximum bond dimension of *M* = 2000 and a convergence threshold of 1.0 × 10^–6^ Ha. Due to the large size of these systems (∼260 electrons and ∼1000 orbitals), the full emb-DMRG-sc-NEVPT2 calculations were not possible for DMRGSCF reference wavefunctions with *M* = 2000. To overcome this limitation, we used the converged orbitals from the *M* = 2000 calculations to initialize DMRGSCF calculations at bond dimensions of 500, 750, and 900. We obtain final energies by extrapolating the DMRGSCF energies to infinite bond dimension and adding the NEVPT2 corrections calculated at *M* = 900 (see Figures S4 and S5), where the latter represents the maximum value that we could use for a full NEVPT2 calculation in all systems studied.

Our emb-DMRGSCF calculations were all initialized using a merging procedure that was previously shown to be reliable for generating initial guesses for emb-CASSCF calculations of reactions on metal clusters.^35^ To do this, we first performed emb-CASSCF calculations using MOLPRO v2024.1.1.^84–88^ We optimized (12e,12o) active spaces for Ni_1_Ag_12_, Pd_1_Ag_12_, and Pt_1_Ag_12_ while we optimized an (11e,11o) active space for Rh_1_Ag_12_ (vide infra). These active spaces were then merged with the optimized (10e,8o) active space from a CASSCF calculation of CO. We used MOKIT^89^ to transfer these orbitals from Molpro to PySCF. The benefit of this approach is that the optimal active space for the metallic cluster can be identified efficiently, leading to an improved guess for much larger emb-DMRGSCF calculations. Moreover, this procedure is shown to be reliable for active spaces up to (22e,20o). Initial testing found that without this merging procedure, PySCF specifically struggled with treating the complex interplay of dopant and host metal orbitals, often favoring the more delocalized Ag 5s orbitals over the dopant orbitals that participate in bond formation with the CO.

## Results

The DFT-projected density of states (Figure 2a) plots for the different SAA models provide valuable initial insights into the nature of the dopant metal d-states. While the Ag(100) host has a clearly identifiable d-band, the dopant metal states give rise to sharp and energetically narrow features below the Fermi level. This reflects earlier literature reports indicating that the mixing of dopant and host metal d-orbitals can be ineffective in SAA systems.^6,12,13^ The dopant d-states are energetically well-separated from the Ag d-band, and the PDOS for Ni-, Pd-, and Pt-doped systems suggest d^10^ configurations for the dopants. Focusing specifically on Pd_1_Ag_12_, the emb-CASSCF (10e,10o) natural orbitals (Figure 2b) reveal that not only is the Pd d^10^ in the SAA, but also that the 4d-orbitals all have equivalent fractional occupation numbers and little hybridization with the Ag host 5s orbitals. This result supports that single-atom Pd has inherently different behavior than Pd in the bulk, which XPS reveals has a d^9^s^1^ configuration.^90^

**Figure 2:**
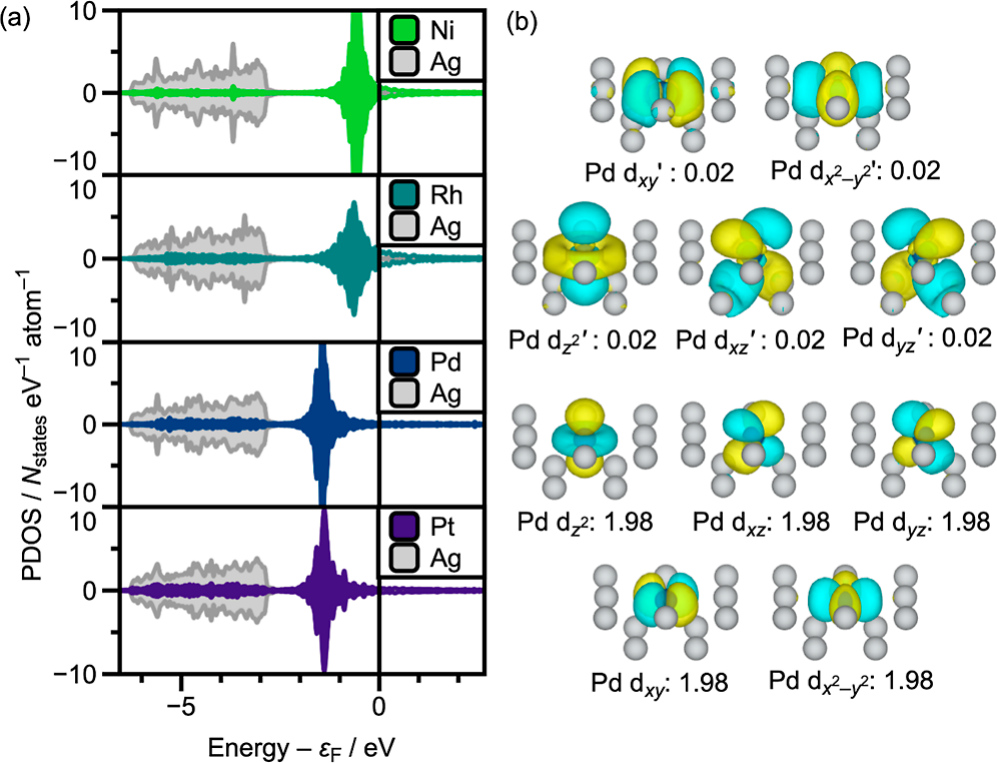
(a) PW-DFT calculated PDOS for Ni-, Rh-, Pd-, and Pt-doped Ag(100) surfaces using five-layer 5 × 5 (Ni, Pd, and Pt) and four-layer 5 × 5 (Rh) slabs where a single dopant replaces a surface layer Ag. With all plots, the states are normalized by the number of each species and as a result the Ag sp-band is obscured by the scale. The Fermi level is set to the zero of energy, represented by the vertical black line. PDOS were obtained using a 9 × 9 × 1 *k*-point mesh with Fermi surface smearing by the tetrahedron method with Blöchl corrections.^91^ (b) emb-CASSCF (10e,10o) natural orbitals for the Pd_1_Ag_12_ cluster. This active space contains all Pd 4d orbitals with equivalent, fractional occupation numbers, which are given in the labels below each isosurface. Orbitals are arranged in all plots left-to-right and bottom-to-top in the order of decreasing occupation number. The orbitals are plotted at a 0.03 Å^–3^ isosurface level.

A natural starting place for studying CO adsorption on the Pd-doped SAA is the largest active space accessible to conventional emb-NEVPT2 based on a CASSCF reference wavefunction, which for this system is (12e,12o). With this active space, only the CO 5σ, 6σ, 1π, 2π*, and Pd 4d orbitals along the bond axis (*z* ^2^, *xz*, and *yz*) can be included in the active space. While (12e,12o) is the largest conventional active space, it represents the absolute minimal model for CO adsorption on Pd_1_Ag_12_. The resulting free energies of CO adsorption are highly negative because of this. With the AVDZ basis set, the ECW approach yields an adsorption free energy of −1.38 eV, which grows to −2.00 eV with the AVTZ basis set, and −2.26 eV once extrapolated^66^ to the CBS limit. This number is concerningly large considering that the adsorption free energy from PW-DFT+D3 is only −1.05 eV. We attribute this behavior to an unequal treatment of the CO adsorbed and desorbed states. Because the Pd 4d-orbitals split when CO adsorbs and the nonbonding d-orbitals have less static correlation in this state, the minimal active space spuriously favors the CO adsorbed state. Calculating the ECW adsorption free energy with a GTO basis for the emb-DFT term in eq (4) , i.e., Δ*G*_ads_^ECW,GTO^, leads to adsorption energies that are approximately 1.5 eV more negative with both AVDZ and AVTZ basis sets (Figure S6). This issue is not related to active space selection and is most likely due to improper cancellation of error.

We undertook systematic expansion of the DMRGSCF active space to understand how including more Pd 4d and some Ag 5s orbitals in our active space would affect the CO adsorption thermodynamics. The first improvement to make is to expand the active space to (16e,16o) by incorporating the nonbonding Pd 4d-orbitals (i.e., *x* ^2^–*y* ^2^ and *xy*) and their correlating orbitals to the (12e,12o) active space. The next logical expansion of the active space would be to (18e,18o) by adding a pair of Ag 5s orbitals to the (16e,16o) active space. By adding the host metal orbitals, we expect to capture some of the coupling between the host and dopant metals. However, an alternative active space with 18 orbitals can be achieved by incorporating the CO 3σ and 4σ in place of the Ag 5s orbitals, which brings the active space to (20e,18o). Lastly, the largest sensible active space of (22e,20o) would incorporate the CO 3σ, 4σ, 5σ, 6σ, 1π, and 2π* orbitals, all Pd 4d-orbitals, and a pair of Ag 5s orbitals (Figure 3a). The Ag orbitals are taken as the highest energy occupied and lowest energy virtual orbitals at the mean-field level and qualitatively represent the orbitals at the Fermi level. Active spaces larger than this would simply incorporate more Ag 5s orbitals, increasing the cost of the calculation with a likely marginal benefit. Indeed, previous ECW studies of CO reduction on copper surfaces^27^ have shown that active spaces containing only (2e,2o) from the metal cluster yield comparable results to active spaces including (4e,4o) and (6e,6o) from the cluster.

**Figure 3:**
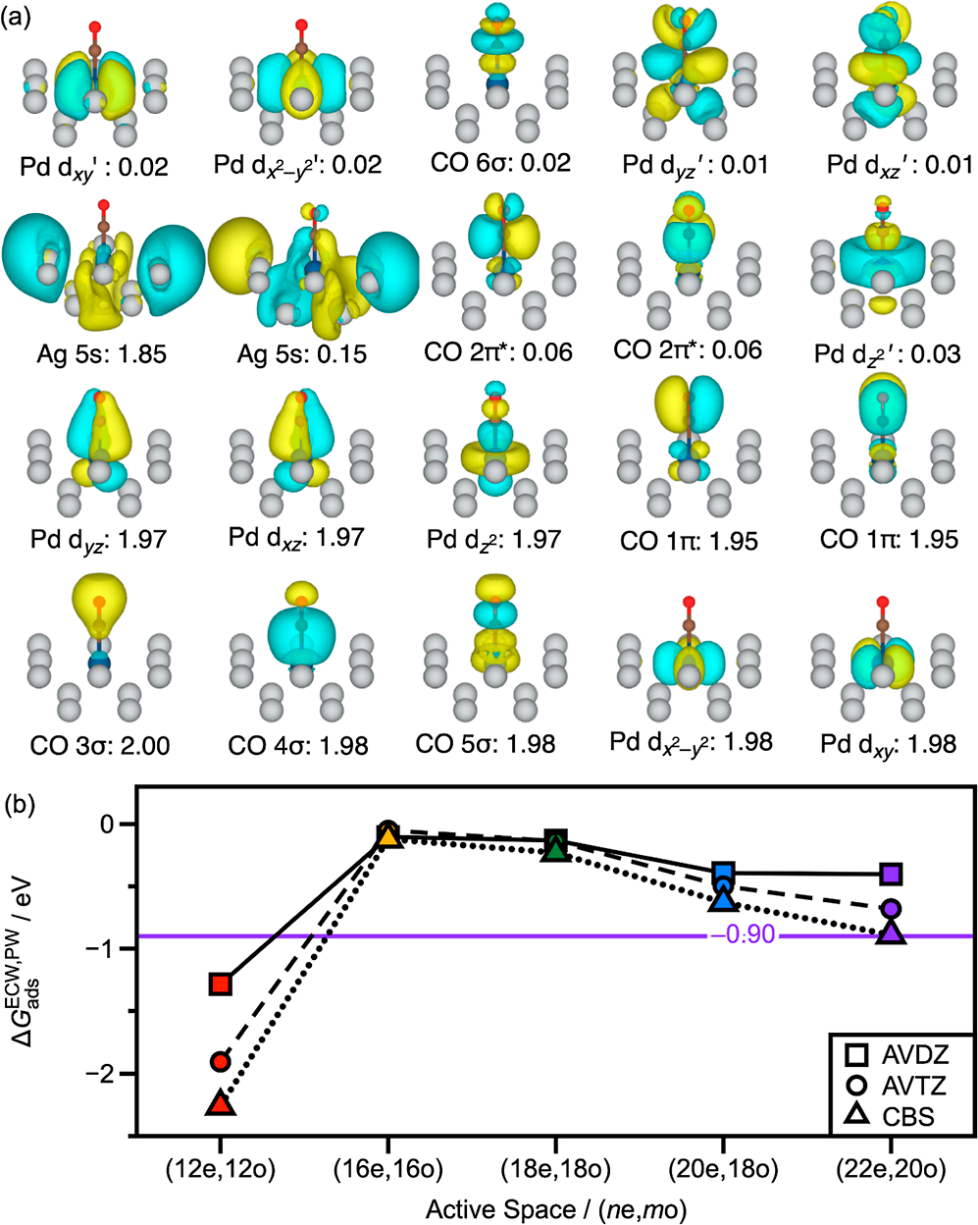
(a) Optimized emb-DMRGSCF (22e,20o) natural orbitals for CO adsorbed on a Pd_1_Ag_12_ cluster arranged in order of decreasing occupation number from left to right, starting from the bottom row. The labels below each orbital isosurface give both the orbital assignment and occupation number. Orbital assignments are based off nodal structure and orbital coefficients. The orbitals are plotted at a 0.03 Å^–3^ isosurface level. (b) ECW adsorption free energy for CO on Pd_1_Ag_12_ as a function of active space size and basis set size with the AVDZ (squares) and AVTZ, (circles) basis sets. We also present results extrapolated to the CBS limit (triangles). Lines connect the points as visual aids. The different active spaces are color coded as red for (12e,12o), yellow for (16e,16o), green for (18e,18o), blue for (20e,18o), and purple for (22e,20o). The purple line and label denote the CO adsorption free energy at the CBS limit with the largest active space of (22e,20o).

With these various large active spaces accessible to emb-DMRG-sc-NEVPT2, we obtain adsorption free energies as a function of the active space size. Incorporating all the Pd d-orbitals with the (16e,16o) active space significantly stabilizes the CO desorbed state relative to the CO adsorbed state, resulting in weaker CO adsorption as shown in Figure 3b. The inclusion of the CO 3σ, 4σ pair with the (20e,18o) active space slightly stabilizes the adsorbed CO relative to the (16e,16o) active space while still being less strongly bound compared to the (12e,12o) active space. At the CBS limit, the (16e,16o) active space yields an adsorption free energy of −0.12 eV while the (20e,18o) active space yields an adsorption free energy of −0.63 eV. Lastly, including a pair of Ag 5s orbitals in the active space has a small stabilizing effect on the adsorption of CO. For the (18e,18o) active space, this is a relatively minor effect, with adsorption free energies being largely unchanged from the (16e,16o) active space. The effect is larger at the (22e,20o) active space, with the adsorption free energy being −0.90 eV at the CBS limit. Regardless, the smaller effect of the Ag 5s orbitals as compared with the Pd d-orbitals is likely another reflection of the poor mixing between the dopant and host metal orbitals.

Turning to the Rh-doped Ag(100) SAA, we find a qualitatively different behavior owing to the d^9^ nature of Rh. With Rh having an odd number of electrons, the minimal active space for CO adsorption is (13e,13o). This active space incorporates the CO 5σ, 6σ, 1π, 2π*, the Rh 4d-orbitals along the bond axis (*z* ^2^, *xz*, and *yz*), and the singly occupied Rh 4d_*xy*_, leaving the $4d_{x^{2}-y^{2}}$ electron pair out of the active space. At this minimal active space, the CO adsorption free energy is −1.97 eV with the AVDZ basis set and −2.20 eV at the CBS limit, close to the adsorption free energy of −2.17 eV at the PW-DFT+D3 level of theory. As with the Pd dopant, we can systematically expand the active space and determine the different adsorption free energies (Figure 4). For Rh, the possible larger active spaces are (15e,15o), (17e,17o), (19e,17o), and (21e,19o). The CO adsorption free energy for the Rh_1_Ag_12_ cluster shows much less dependence on the active space size than was seen for the Pd_1_Ag_12_ cluster, as shown in Figure 4. The (15e,15o) active space, which adds the $4d_{x^{2}-y^{2}}$ electron pair to the set of orbitals in the (13e,13o) active space, yields approximately the same results as the minimal active space, with an adsorption free energy of −2.02 eV with the AVDZ basis set and −2.17 eV at the CBS limit. This lower degree of change relative to a similar change in the active space for Pd_1_Ag_12_ is because the minimal active space already incorporates most of the Rh d-orbitals, including the 4d_*xy*_, which couples to the Ag 5s states at the Fermi level; moreover, the added orbital pair does not mix with the CO orbitals. Adding a pair of Ag 5s orbitals to form the (17e,17o) active space yields results identical to those of the (15e, 15o) case with the AVDZ basis set. The most negative adsorption free energy at the CBS limit is −2.38 eV (−2.21 eV with the AVDZ basis set) and is found with the (19e,17o) active space (which incorporates all Rh 4d-orbitals plus the CO 3σ, 4σ, 5σ, 6σ, 1π, and 2π* orbitals). With the largest active space of (21e,19o), which adds pair of Ag 5s orbitals to the set contained in the (19e,17o) active space (Figure S7), the adsorption free energy is −2.19 with the AVDZ basis set and −2.29 eV at the CBS limit. We find that calculating the ECW adsorption free energy with an emb-GTO-DFT calculation (i.e., Δ*G*_ads_^ECW,GTO^) yields quantitatively similar results for AVDZ and AVTZ basis sets (Figure S8) and the same results at the CBS limit. We attribute this weaker dependence on active space size to the minimal active space being forced to include a larger proportion of the Rh 4d-orbitals by virtue of the singly occupied 4d_*xy*_ such that the minimal active space presents a more balanced treatment of the CO adsorbed and desorbed states than the minimal active space for the Pd dopant.

**Figure 4:**
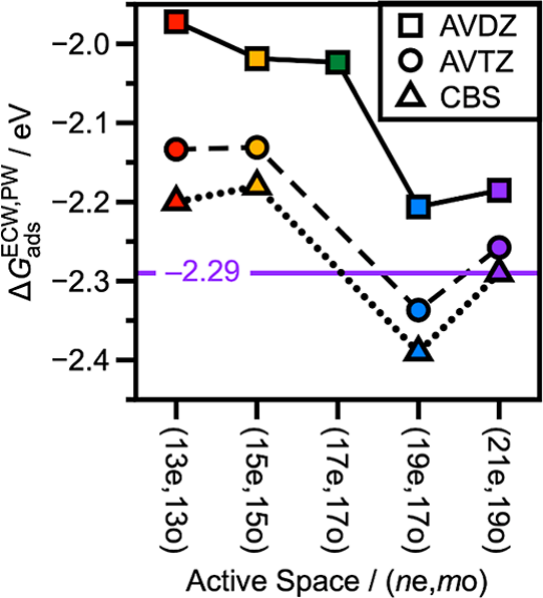
ECW adsorption free energy for CO on Rh_1_Ag_12_ as a function of active space size and basis set size with the AVDZ (squares) and AVTZ (circles) basis sets. We also present results extrapolated to the CBS limit (triangles). Lines connect the points as visual aids. The same color-coding scheme as in Figure 2 is retained and the horizontal axis marks denote the active space. The TZ point for the (17e,17o) active space is omitted because we were unable to obtain a set of natural orbitals consistent with the natural orbitals from the DZ basis set when CO is adsorbed on Rh_1_Ag_12_. The purple line and label denote the CO adsorption free energy at the CBS limit with the largest active space of (21e,19o).

The Pt-dopant represents a system where being restricted to smaller active spaces forces one to choose between keeping the active orbitals consistent between the CO adsorbed and desorbed states or always using the lowest energy wavefunction. To understand how this arises, it is best to start with the Pt_1_Ag_12_ cluster and the optimal (12e,12o) active space at the emb-NEVPT2 and emb-DMRG-NEVPT2 levels. With both AVDZ and AVTZ basis sets, this active space contains all of the Pt 5*d*/6d orbitals and a pair of Ag 5s orbitals (Figures S9 and S10). At the emb-CASSCF/emb-DMRGSCF level, it is possible to find a slightly lower energy active space, which includes a Pt 6*s*/7s pair in place of the Ag 5s pair (Figure S11). In the minimal (12e,12o) active space for a calculation containing CO and the Pt_1_Ag_12_ cluster, the cluster can only contribute (6e,6o). For the CO adsorbed state, this includes Pt 5d_*z*_^2^, 5d_*xz*_, and 5d_*yz*_ as well as their correlating orbitals. When CO is desorbed, the Pt 5d_*z*_^2^ is replaced by the Pt 5 $d_{x^{2}-y^{2}}$ in the lowest energy active space at both emb-CASSCF and emb-NEVPT2 levels (Figure S12). In this active space, the Pt 5d_*xz*_, 5d_*yz*_, and 5 $d_{x^{2}-y^{2}}$ have occupation numbers of 1.97, 1.97, and 1.96 respectively with the AVDZ basis set (Figure S12), and the AVTZ basis set yields similar occupation numbers. It is possible to retain the Pt 5d_*z*_^2^ in the active space, in which case the Pt 5d_*xz*_, 5d_*yz*_, and 5d_*z*_^2^ all have occupation numbers of 1.99, but with the AVDZ and AVTZ basis sets, the resulting active space is ∼2.0 eV higher in energy at the emb-DMRG-NEVPT2 level. Thus, at the minimal active space, a choice must be made to either use the lower-energy wavefunction for the CO desorbed state or to use the one that keeps the active orbitals consistent with the CO adsorbed state.

To distinguish between these two possible minimal active spaces for desorbed CO in computed adsorption free energies, we will denote values calculated using the lowest-energy active space for both CO adsorbed and desorbed as (12e,12o) and the values calculated by maintaining the same active orbitals for both CO adsorbed and desorbed as (12e,12o)*. For the (12e,12o) active space, we find that the CO adsorption free energy is 0.46 eV for the AVDZ basis, 0.35 eV for the AVTZ basis set, and 0.30 eV at the CBS limit. With the (12e,12o)* active space, we predict the CO adsorption energy to be −1.53 eV for the AVDZ basis set, −1.63 eV for the AVTZ basis set, and −1.66 eV at the CBS limit. The predicted CO adsorption free energy clearly depends highly on the chosen minimal active space.

The concern of choosing between the lowest-energy description of desorbed CO and the continuity of active orbitals naturally disappears at the larger active spaces accessible to emb-DMRG-NEVPT2. The first larger active space of (16e,16o) incorporates all Pt 5d-orbitals in addition to the CO 5σ, 6σ, 1π, and 2π* orbitals. At this active space, the adsorption free energy is +0.87 eV for the AVDZ basis set and −1.19 eV for the AVTZ basis set. This large discrepancy is alarming, but it does indicate that it is critical to maintain a consistent set of active orbitals as described above. The origin of the discrepancy between basis sets will be discussed further below (vide infra). If we expand the active space to (18e,18o), we incorporate a pair of Ag 5s orbitals, which yields CO adsorption free energies of +1.15 eV for the AVDZ basis set and −1.25 eV for the AVTZ basis set. As with Pd, the adsorbed CO is further stabilized when the CO 3σ and 4σ are included in the active space, with the (20e,18o) active space yielding adsorption free energies of +0.42 eV for the AVDZ basis set and −1.60 eV for the AVTZ basis set. Lastly, the largest active space of (22e,20o) (Figure S13) predicts adsorption free energies of +0.66 eV with the AVDZ basis set and −1.67 eV with the AVTZ basis set (purple line Figure 5a), the latter of which is close to the CBS extrapolated value using the (12e,12o)* active space. The agreement between the AVTZ calculations with the (22e,20o) active space and the CBS limit with the (12e,12o)* active space confirms that (12e,12o)* is the correct choice for the minimal active space and also highlights the importance of performing calculations with basis sets of increasing size.

**Figure 5:**
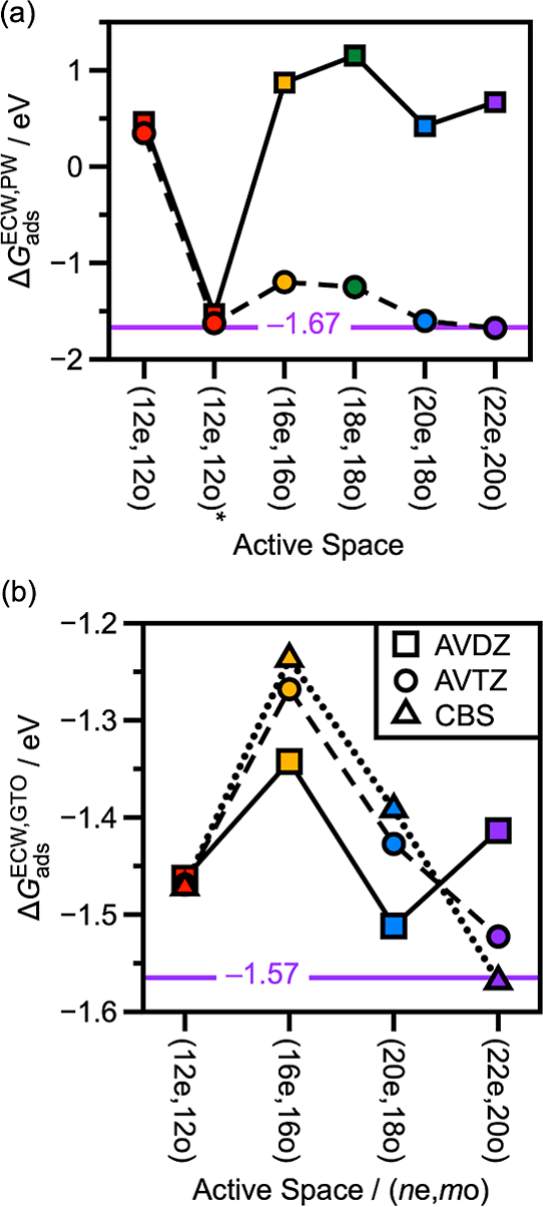
ECW adsorption free energies for CO on (a) Pt_1_Ag_12_ and (b) Ni_1_Ag_12_ as a function of active space size and basis set size with the aug-cc-pVDZ (AVDZ, squares) and aug-cc-pVTZ (AVTZ, circles) basis sets, and extrapolation to the CBS limit (triangles). Lines serve as visual aids. The different active spaces are color-coded as red for (12e,12o), yellow for (16e,16o), green for (18e,18o), blue for (20e,18o), and purple for (22e,20o). The purple lines and labels give the value of the adsorption free energy with the (22e,22o) active space and the AVTZ basis set in (a) and at the CBS limit in (b).

In active spaces containing all Pt 5d/6d orbitals, the discrepancies between adsorption free energies calculated with AVDZ and AVTZ basis sets originate in the changes in the multireference character of the Pt 5d/6d orbitals. For the CO adsorbed state, the occupation numbers across all active spaces remain relatively unchanged between the AVDZ and AVTZ basis sets. For desorbed CO, however, the occupation of the Pt 5d orbitals changes significantly between basis sets for the active spaces containing all Pt 5d/6d orbitals. With the AVDZ basis set, the Pt 5d orbitals all have occupation numbers of 1.98 or lower when CO is desorbed (Figure S9), which is similar to the occupation numbers of lowest-energy minimal active space for desorbed CO in both AVDZ and AVTZ basis sets. With the AVTZ basis set, the Pt 5d orbitals all have occupation numbers of 1.99 (Figure S10) when CO is desorbed, which is also observed in the higher energy (12e,12o)* active space (vide supra). Moreover, the adsorption free energies for the large active spaces at the AVDZ basis set are close to the adsorption free energies for the (12e,12o) active space, while the adsorption free energies for the large active spaces at the AVTZ basis set are close to the adsorption free energies with the (12e,12o)* active space. Because the only difference between the (12e,12o) and (12e,12o)* active spaces is the treatment of the desorbed CO, we attribute differences of the predicted adsorption free energies at large active spaces between AVDZ and AVTZ basis sets to a change in the description of the desorbed CO state. Intriguingly, the discrepancy between the basis sets containing diffuse functions originates in the emb-DMRG-NEVPT2 corrections, as the emb-DMRGSCF contribution to the adsorption free energy is roughly equivalent between AVDZ and AVTZ basis sets for all active spaces, e.g., being +5.10 eV for the AVDZ and +5.06 for the AVTZ basis sets with the (16e,16o) active space, with the contributions from PW-DFT being consistent across the two basis sets.

With the Pt dopant, we also tested the effect of removing the diffuse functions from the basis set by performing calculations with the (16e,16o) active space and the cc-pVDZ and cc-pVTZ basis sets. With the cc-pVDZ basis set, we were unable to obtain a consistent set of active orbitals between the CO adsorbed and the desorbed states. With the CO desorbed state, we were able to obtain a set of active orbitals that were consistent with the AVDZ results. For the adsorbed state, however, a Pt 6s/7s pair and an Ag 5s pair replace the Pt 5d_*xy*_ and 5 $d_{x^{2}-y^{2}}$ (and their correlating orbitals) in the active space, which is inconsistent with the AVDZ results. While the ECW adsorption free energy is lower with the cc-pVDZ basis set (−0.74 eV) than with the AVDZ basis set (+0.87 eV), this decrease is likely spurious due to inconsistencies in the active spaces between the CO adsorbed and desorbed states. Both the CO adsorbed and desorbed (16e,16o) active spaces with the cc-pVTZ basis set were consistent with the corresponding active spaces with the AVTZ basis set, leading to a nearly identical ECW adsorption free energy of −1.24 eV. Notably, the triple-ζ basis set results differ only by 0.05 eV with the removal of diffuse functions from the basis set.

We do not attempt basis set extrapolation for these large active spaces due to the large changes between AVDZ and AVTZ basis sets, and calculations with a quadruple-ζ basis set are prohibitively expensive. However, given the consistency between the (12e,12o)* results at the CBS limit and the AVTZ results with the (22e,20o) active space, we estimate the CO adsorption free energy to be −1.67 eV. Calculations with the emb-GTO-DFT contribution are given for comparison in Figure S14. Ultimately, the access to the larger active spaces provided by emb-DMRGSCF and emb-DMRG-NEVPT2 eliminates the choice that must be made for the minimal active space and allows a clear prediction of the CO adsorption free energy.

Lastly, the Ni-dopant sees significant population exchange occurring upon CO desorption. It is again useful to begin by focusing on the embedded Ni_1_Ag_12_ with a (12e,12o) active space (Figure S15). In the absence of the CO, the Ni_1_Ag_12_ is an open-shell singlet, with the Ni 3d_*xy*_ and 4s both being singly occupied and the FCI expansion dominated by two determinants where either the 3d_*xy*_ or 4s are doubly occupied. When the CO is present in the calculation but desorbed from the SAA by 12 Å, the triplet spin state is stabilized relative to the open-shell singlet, with the 3d_*xy*_ and 4s each being singly occupied. As the CO adsorbs, 3d_*xy*_ becomes doubly occupied, which leads to a singlet spin-state being the most favorable.

Of all the systems tested, the CO adsorption free energies for the Ni dopant exhibit the least dependence on the active space size. In addition to the CO 5σ, 6σ, 1π, and 2π*, the minimal active space for Ni of (12e,12o) incorporates the Ni 3d_*xz*_, 3d_*yz*_, and 3d_*z*_^2^ when CO is adsorbed and the Ni 3d_*xz*_, Ni 3d_*z*_^2^, 3d_*xy*_ and 4s when CO is desorbed. This leads to a CO adsorption free energy of −1.48 eV with the AVDZ basis set and also at the CBS limit, 0.15 eV less negative than the −1.63 eV predicted at the PW-DFT+D3 level of theory. As with Pt, a (16e,16o) active space incorporates all of the Ni 3d-orbitals when CO is adsorbed and reveals that the Ni 3d-orbitals all have fractional occupations lower than 1.98, confirming that they are important to include in the active space. However, the (16e,16o) active space does not greatly weaken the CO adsorption free energy, yielding a value of −1.34 eV with the AVDZ basis set and −1.24 eV at the CBS limit. As with the Pd and Pt dopants, including the CO 3σ, 4σ pair in the active space (i.e., 20e,18o) stabilizes CO adsorption relative to the (16e,16o) active space with an adsorption free energy of −1.52 eV with the AVDZ basis set and −1.39 at the CBS limit. Lastly, a (22e,20o) active space (Figure S16) captures Ag 5s and Ni 3*d*/4s coupling that was important in Pt and Pd but has an overall minor effect here. With this active space, the adsorption free energies are −1.42 eV with the AVDZ basis set and −1.57 at the CBS limit (purple line, Figure 5b), which is not significantly different from the prediction of the minimal active space. Results with emb-PW-DFT are given in Figure S17.

## Conclusions

In this work, we have combined our group’s DFET/ECW methods with DMRGSCF and DMRG-NEVPT2 for a reliable multireference treatment of chemistry on metallic surfaces when large active spaces are required. We have used these methods to understand the adsorption of CO on a range of catalytically relevant SAAs based on a Ag host metal. Through these methods, we have systematically expanded the active spaces to gauge how the active space selection affects the CO adsorption free energy.

We find that ECW methods predict adsorption free energies that differ from predictions at the PW-DFT+D3 level by 0.10–0.27 eV. Both DFT and ECW methods predict Rh as adsorbing CO the most strongly and Pd as adsorbing CO the most weakly, although this is not surprising given the large difference in their adsorption free energies at both levels of theory. However, ECW methods predict that CO adsorbs more strongly on Pt than on Ni, which is the opposite of what DFT predicts, providing resolution when the differences in the adsorption free energies are smaller.

Our studies reveal that the dopant metal d-orbitals cannot be thought of as akin to those of an isolated atom, because coupling between the Ag 5s and dopant metal d-orbitals is important to describing CO adsorption in all cases. For all systems studied, the minimal active space is the largest active space that can be handled by conventional methods. In the Pd system, the minimal active space describes a highly overbound CO because it treats the CO adsorbed state preferentially to the CO desorbed state. This preferential treatment is itself related to the splitting of the dopant d-states in the presence of CO, where the d-orbitals oriented along the bond axis are less occupied than nonbonding d-orbitals. The adsorption free energies for the Rh and Ni dopants depend more weakly on active space size, but with both, inclusion of the CO 3σ/4σ pair and Ag 5s orbitals is necessary when all dopant d-orbitals are included in the active space. With Pt, the minimal active space forces a choice between using the lowest-energy active spaces and those that maintain consistent orbitals across the reaction coordinate. However, when all Pt d-orbitals are included in the active space, this concern disappears. This has important implications for future multireference work on SAAs. First, in dopants with full or nearly full d-shells, even the nonbonding d-orbitals can be important to an accurate description of the chemistry. Second, the host metal orbitals are important to the chemistry even when the bond formation/scission seemingly only involves the dopant site, and these issues are likely to be exacerbated when both dopant and host metal sites form bonds with adsorbed species.

## Supplementary Material



## Notes

ZPE and entropy corrections for free energies; isosurface plots of embedding potentials; description of DMRGSCF energy extrapolation procedure; and additional data and large active space natural orbitals for Pd-, Rh-, Pt-, and Ni-dopants (PDF)
