# Correction to “Combining Density Functional Embedding Theory and DMRG-NEVPT2 to Treat Large Active Spaces: Addressing Electronic Structure Complexity in Single-Atom Alloys”

**DOI:** 10.1021/acs.jctc.6c00911

**Published:** 2026-06-02

**Authors:** Phillips Hutchison, Ziyang Wei, Emily A. Carter

We write to report three numerical errors we discovered in our recent paper, none of which change the analysis or conclusions of our work, but to correct the record, we report them here. We also report one mislabeled active space in a figure caption in the Supporting Information (SI) file.

First, we identified a minor error in the reported adsorption free energies for Pd- and Ni-doped single-atom alloys (SAAs) at the planewave density functional theory with dispersion corrections (PW-DFT+D3) level. The adsorption free energies reported in the original publication were taken from a smaller five-layer 4 × 4 supercell slab model as opposed to the stated five-layer 5 × 5 supercell slab. The PW-DFT-D3 adsorption free energies for the five-layer 5 × 5 slab differ from the reported values by 0.06 eV. Specifically, the CO adsorption free energy at the PW-DFT+D3 level changes from −1.05 eV to −1.11 eV for the Pd dopant (reported in the second paragraph of the Results section) and from −1.63 eV to −1.69 eV for the Ni dopant (reported in last paragraph of the Results section). (The analogous value for the Pt dopant was inadvertently not reported; for completeness, it is −1.47 eV for the five-layer 5 × 5 slab model.) *Importantly, these changes do not affect the reported embedded correlated wave function (ECW) adsorption free energies, as the full slab PW-DFT contributions for those calculations were taken from the correct five-layer 5 × 5 supercell slab models.*

Second, we identified an error in our calculation of the ECW adsorption free energies for one case: Rh-doped Ag(100) when treating the embedded cluster at the Gaussian-type orbital (GTO) DFT level with the aug-cc-pVDZ (AVDZ) basis set. Our original plotting file converted between *E* ^ECW,PW^ and *E* ^ECW,GTO^, which differ only in the emb-DFT contributions, as seen in eqs 1 and 2 below. Equation 3 provides the means to convert from the PW result to the GTO result.

1 $$E^{\mathrm{ECW,GTO}}=E_{\mathrm{slab}}^{\mathrm{PW‐DFT}}+E_{\mathrm{cluster}}^{\mathrm{emb‐CW}}-E_{\mathrm{cluster}}^{\mathrm{emb‐GTO‐DFT}}$$

2 $$E^{\mathrm{ECW,PW}}=E_{\mathrm{slab}}^{\mathrm{PW‐DFT}}+E_{\mathrm{cluster}}^{\mathrm{emb‐CW}}-E_{\mathrm{cluster}}^{\mathrm{emb‐PW‐DFT}}$$

3 $$E^{\mathrm{ECW,GTO}}=E^{\mathrm{ECW,PW}}+E_{\mathrm{cluster}}^{\mathrm{emb‐PW‐DFT}}-E_{\mathrm{cluster}}^{\mathrm{emb‐GTO‐DFT}}$$

*E*_slab_^PW‑DFT^ is the energy of the periodic slab at the PW-DFT level, *E*_cluster_^emb‑CW^ is the energy of the embedded cluster at the correlated wave function (CW) level, *E*_cluster_^emb‑PW‑DFT^ is the energy of the embedded cluster at the PW-DFT level, and *E*_cluster_^emb‑GTO‑DFT^ is the energy of the embedded cluster at the GTO-DFT level. With Rh and the AVDZ basis set, a sign error in our original file led to *E* ^ECW,GTO^ being calculated as *E* ^ECW,GTO^ = *E* ^ECW,PW^ – *E*_cluster_^emb‑PW‑DFT^ + *E*_cluster_^emb‑GTO‑DFT^, which subsequently affected the ECW adsorption free energies. *This sign error only affected the AVDZ values of* Δ*G*_ads_^ECW,GTO^ *(eq 8 in the original paper) of the Rh-doped system (all other systems/bases were double-checked for correctness), plotted in* Figure S8, *which were 0.33 eV too positive in the original figure. The corrected values of* Δ*G*_ads_^ECW,GTO^ *with the AVDZ basis set (revised* Figure S8 *) are now in closer agreement with the* Δ*G*_ads_^ECW,PW^ *values reported in the main text and the* Δ*G*_ads_^ECW,GTO^ *values with the AVTZ basis set*. *The change in the AVDZ values of* Δ*G*_ads_^ECW,GTO^ *of course also affects the complete basis set (CBS) extrapolation: the corrected extrapolated value of* Δ*G*_ads_^ECW,GTO^ *changes from −2.5 eV to −2.29 eV with the (21e,19o) active space, which is now coincidentally but pleasingly the exact same value as* Δ*G*_ads_^ECW,PW^ *at the CBS limit for the same active space.*

Third, the embedding potential isosurface values in the captions for Figure 1 and Figures S1–S3 in the SI were reported incorrectly. In our original calculation, we multiplied the plotted isosurface value by the unit cell volume in Å^–3^ instead of Bohr^–3^. As a result, the isosurface values are 6.75 times too low in the original figure captions. The correct *V*_emb_ isosurface values are ±0.88 V for Pd_1_Ag_12_ and Ni_1_Ag_12_, ±1.05 V for Pt_1_Ag_12_, and ±0.50 V for Rh_1_Ag_12_. *This scaling error only affected reporting of which isosurface was plotted but has no effect on the actual results reported.*

Lastly, the caption for Figure S15 in the SI mistakenly identified the active space as (12e,12o) when it actually corresponds to (10e,10o), as is readily apparent from the figure itself.

A corrected Supporting Information file has been provided to replace the original file.

## Supplementary Material



## Notes

ZPE and entropy corrections for free energies, isosurface plots of embedding potentials, description of DMRGSCF energy extrapolation procedure, additional data and large active space natural orbitals for Pd-, Rh-, Pt-, and Ni- dopants (PDF)

