# Supplementary material for: Combining Density Functional Embedding Theory and DMRG-NEVPT2 to Treat Large Active Spaces: Addressing Electronic Structure Complexity in Single-Atom Alloys
Source: J Chem Theory Comput. 2026 Feb 19;22(5):2313–23. doi: 10.1021/acs.jctc.5c02119 (PMC12980710; doi:10.1021/acs.jctc.5c02119)
Supplement: Supplementary file 1 [file ct5c02119_si_001.pdf]

Supporting Information for Combining Density Functional Embedding Theory and DMRG-NEVPT2 to Treat Large Active Spaces: Addressing Electronic Structure Complexity in Single-Atom Alloys

Phillips Hutchison[1], Ziyang Wei[1], and Emily A. Carter[1,2]*

[1]Department of Mechanical and Aerospace Engineering, Princeton University, 41 Olden St., Princeton, NJ, 08544
[2] Andlinger Center for Energy and the Environment and Program in Applied and Computational Mathematics, Princeton University, Princeton, New Jersey 08544-5263, United States

*Corresponding Author: eac@princeton.edu

Table of Contents

Table S1. 2
Optimized Embedding Potentials for Rh-, Pt-, and Ni-Doped Ag(100) 2
Obtaining emb-DMRG-sc-NEVPT2 Energies at Large Bond Dimension 3
CO Adsorption Free Energy on Pd_1Ag_{12} with Emb-GTO-DFT 6
Additional Data for CO on Rh_1Ag_{12} 6
Additional Data for CO on Pt_1Ag_{12} 8
Additional Data for CO on Ni_1Ag_{12} 13

Table S1. Zero-Point Energy (ZPE) and Entropic (*TS*) Corrections for SAA Models in eV.

Dopant	ZPE(CO_{ads})	*TS*(CO_{ads})	ZPE(CO_{des})	*TS*(CO_{des})
Ni	0.36	1.44	0.30	1.76
Rh	0.37	1.41	0.30	1.75
Pd	0.35	1.43	0.30	1.73
Pt	0.36	1.44	0.30	1.75

Optimized Embedding Potentials for Rh-, Pt-, and Ni-Doped Ag(100)

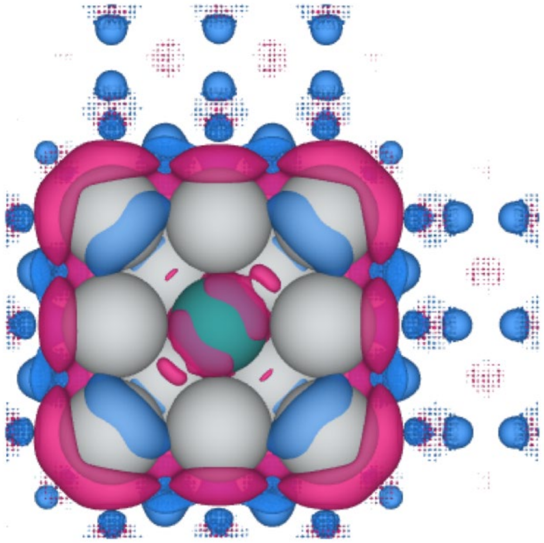

Figure S1. Optimized embedding potential V_{emb} for Rh_1Ag_{12}. The blue regions correspond to +0.08 V and the pink regions are –0.08 V. The Rh is shown in teal and the Ag in grey.

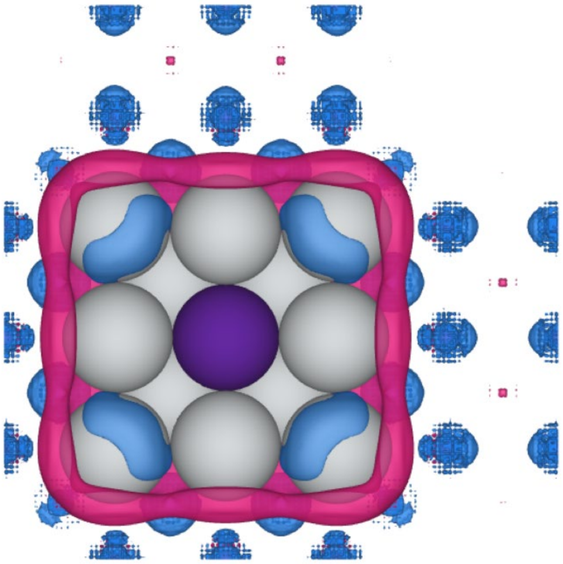

Figure S2. Optimized embedding potential V_{emb} for Pt_1Ag_{12}. The blue regions correspond to +0.16 V and the pink regions are –0.16 V). The Pt is shown in purple and the Ag in grey.

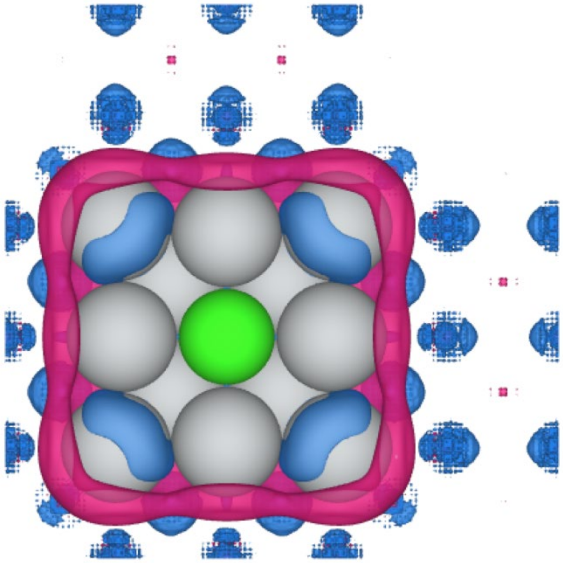

Figure S3. Optimized embedding potential V_{emb} for Ni_1Ag_{12}. The blue regions correspond to +0.14 V and the pink regions are –0.14 V. The Ni is shown in lime green and the Ag in grey.

Obtaining emb-DMRG-sc-NEVPT2 Energies at Large Bond Dimension

We calculate the DMRGSCF adsorption energy as $\Delta E^{\text{emb–DMRGSCF}} = E^{\text{emb–DMRGSCF}}(CO_{ads}) - E^{\text{emb–DMRGSCF}}(CO_{des})$ and extrapolate it to large M[1, 2] using a simple linear fit to adsorption energy plotted versus $1/M$. The calculations at smaller bond dimensions are initialized using the converged natural orbitals of a DMRGSCF calculation with $M = 2000$.

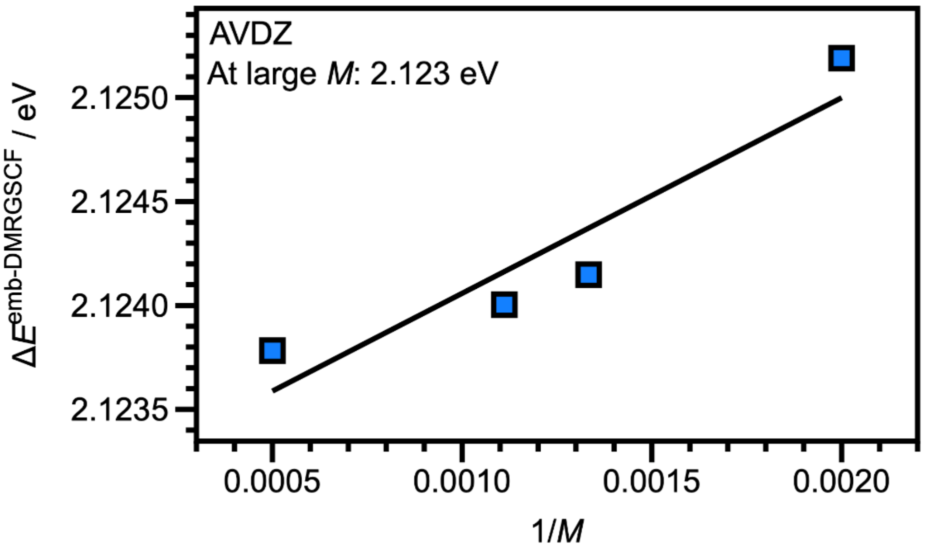

Figure S4. Extrapolation scheme for emb-DMRGSCF adsorption energies at large bond dimensions applied to CO adsorption on Pd_1Ag_{12} with an aug-cc-pVDZ basis set. The full ECW adsorption energy would also require contributions from periodic PW-DFT and embedded cluster DFT calculations. To obtain the adsorption energy contribution from emb-DMRGSCF at large bond dimension, we use a linear fit to the emb-DMRGSCF adsorption energy values calculated a $M = 500$, 750, 900, and 2000 plotted against $1/M$.

We tested the effect of calculating the NEVPT2 contributions using DMRGSCF reference wavefunctions with $M = 500$, 750, 900. Our findings indicate that while in some cases, the NEVPT2 contribution may extrapolate linearly with $1/M$, sufficient accuracy can be achieved with a single calculation at $M = 900$. At worst, using this approach is expected to introduce 30 meV of error for the largest active spaces with an aug-cc-pVTZ basis set (Figure S5). We made this assessment by comparing the adsorption free energies obtained by extrapolating *both* the

DMRGSCF and NEVPT2 energies to infinite bond dimension versus extrapolating *only* the DMRGSCF energy to infinite bond dimension and evaluating the NEVPT2 energy from a DMRGSCF reference wavefunction with a bond dimension of 900. This level of error is much lower than the error introduced by using the wrong active space.

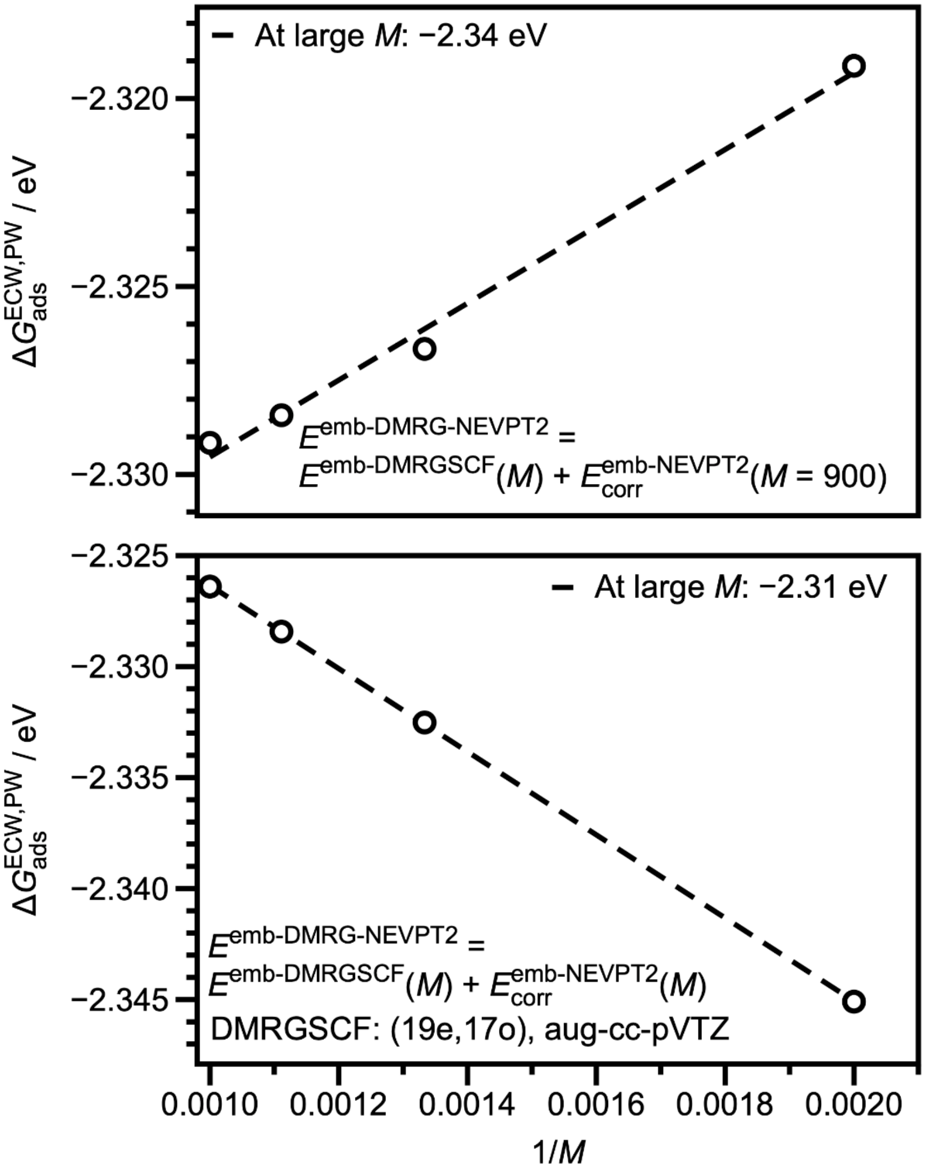

Figure S5. Comparison between ECW adsorption energies of CO on Rh_1Ag_{12} where the emb-DMRG-NEVPT2 contribution is obtained by extrapolating to large bond dimensions with NEVPT2 correlation energies obtained for multiple bond dimensions (bottom) or only a single bond dimension (top). In the bottom plot, the DMRG-NEVPT2 corrections are calculated for DMRGSCF reference wavefunctions obtained with M = 500, 750, 900, and 1000. In the top plot,

the emb-DMRGSCF energies are calculated at M = 500, 750, 900, and 1000 and the NEVPT2 corrections are only obtained from the calculation at M = 900. The active space for these calculations was (19e,17o) and an aug-cc-pVTZ basis set was used. Extrapolated adsorption energies are listed on the respective plots and linear fits are represented by the dashed lines.

CO Adsorption Free Energy on Pd_1Ag_{12} with Emb-GTO-DFT

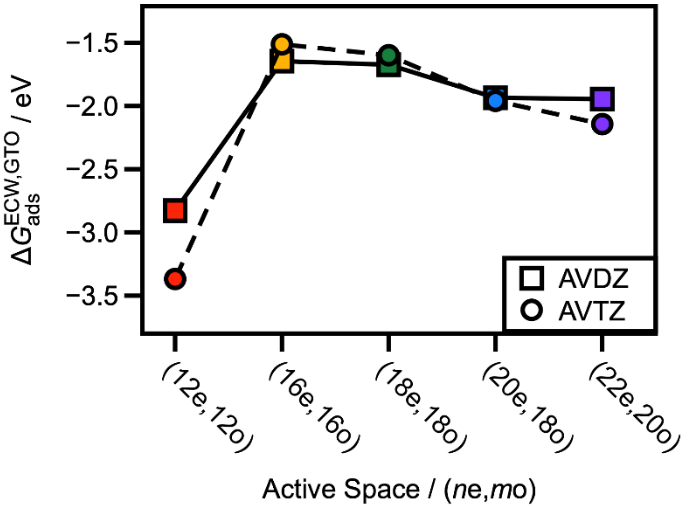

Figure S6. Adsorption free energy for CO on Pd_1Ag_{12} as a function of active space size and basis set size using a Gaussian-type orbital (GTO) basis set for the emb-DFT term in the ECW expression. Compare to Figure 3b in the main text.

Additional Data for CO on Rh_1Ag_{12}

Here we show the emb-DMRGSCF optimize natural orbitals for the largest considered active spaces for CO adsorbed on Rh_1Ag_{12} (Figure S7), The largest active space is (21e,19o) and that active space incorporates the CO 3σ, 4σ, 5σ, 6σ, 1π, and 2π orbitals, all the Rh 4d orbitals, and a pair of Ag 5s orbitals. The Rh–CO π/π* orbitals were assigned based both on nodal symmetry and through the analysis of natural orbital coefficients.

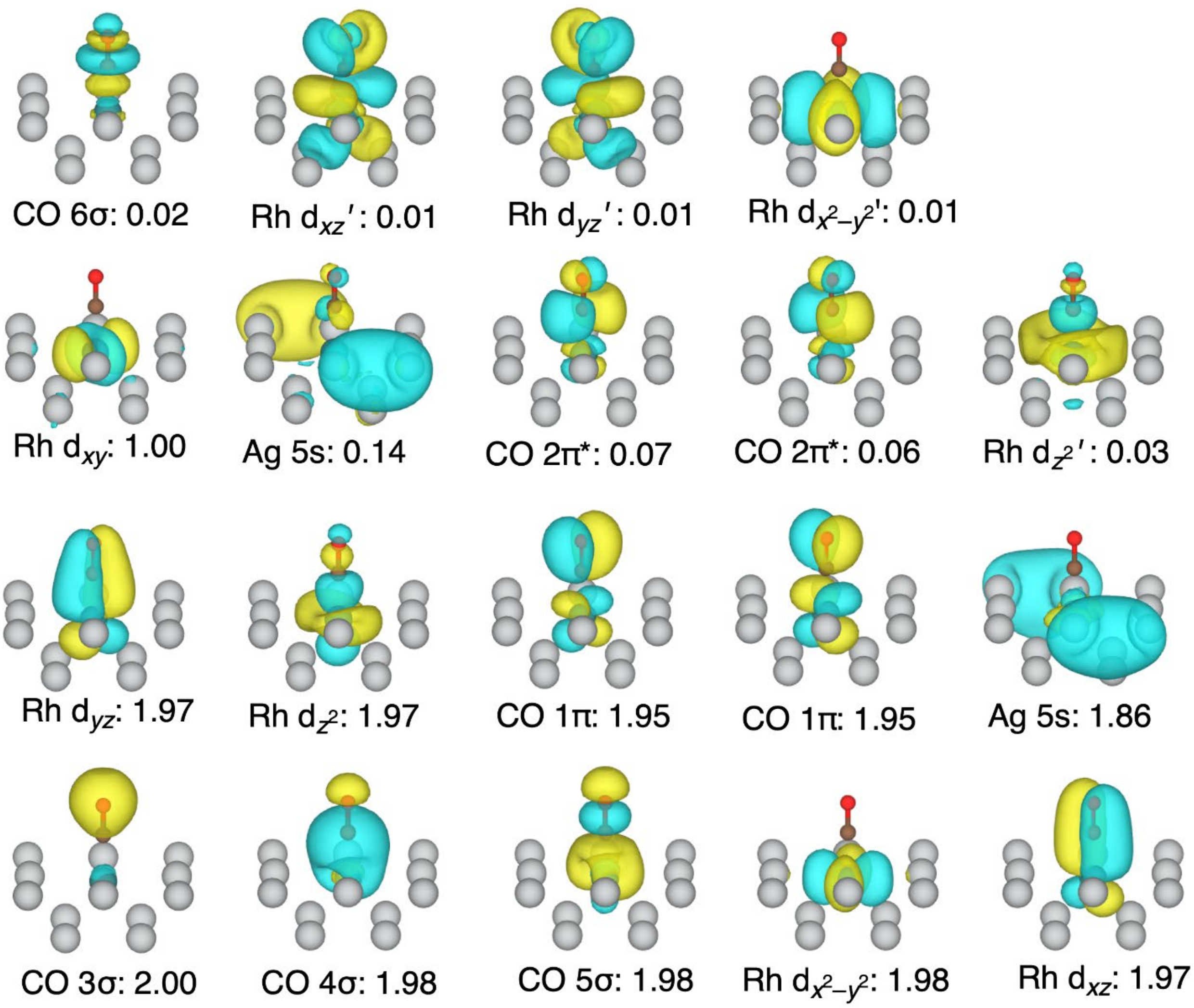

Figure S7. Emb-DMRGSCF(21e,19o) natural orbitals for CO adsorbed on Rh_1Ag_{12} calculated with an aug-cc-pVDZ basis set and a maximum bond dimension of 2000. The orbitals are arranged in order of decreasing occupation number from left to right, starting from the bottom row. The labels below each orbital isosurface give both the orbital assignment and occupation number. The orbitals are plotted at a 0.03 Å^{-3} isosurface level.

For CO adsorption on Rh_1Ag_{12}, using a GTO basis set for the emb-DFT calculation yields quantitatively similar results to the calculations employing a PW-basis set (Figure S8).

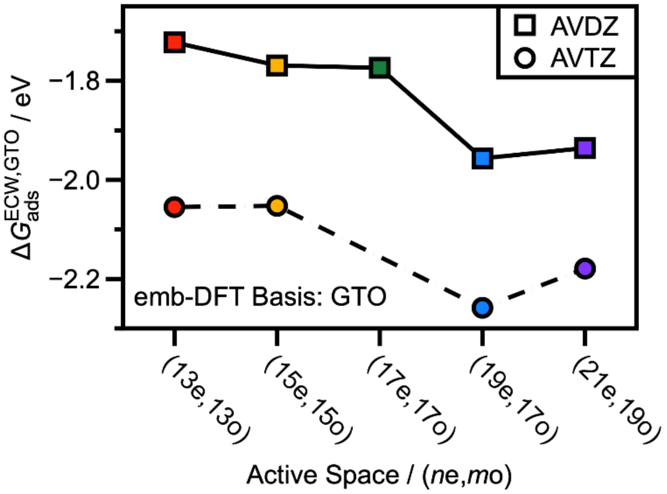

Figure S8. Adsorption free energy for CO on Rh_1Ag_{12} as a function of active space size and basis set size using a GTO basis set for the emb-DFT term in the ECW expression. Compare to Figure 4 in the main text.

Additional Data for CO on Pt_1Ag_{12}

Here, we show the optimized natural orbitals for the embedded Pt_1Ag_{12} cluster in the absence of any CO. Note that when the desorbed CO is present in a supermolecule approach, the active spaces grow to (22e,20o), but the metal cluster's orbitals are unaffected. There are two possible active spaces that incorporate all the Pt 5d orbitals, one that incorporates a Ag 5s pair (Figure S9-10) and one that incorporates a Pt 6s/7s pair (Figure S11). Both active spaces show equivalent occupation of the Pt 5d orbitals for the AVDZ basis set, but the active space that incorporates the Ag 5s pair is lower in energy at the emb-DMRG-NEVPT2 level by –0.65 eV for the AVDZ basis set and –1.44 eV for the AVTZ basis set.

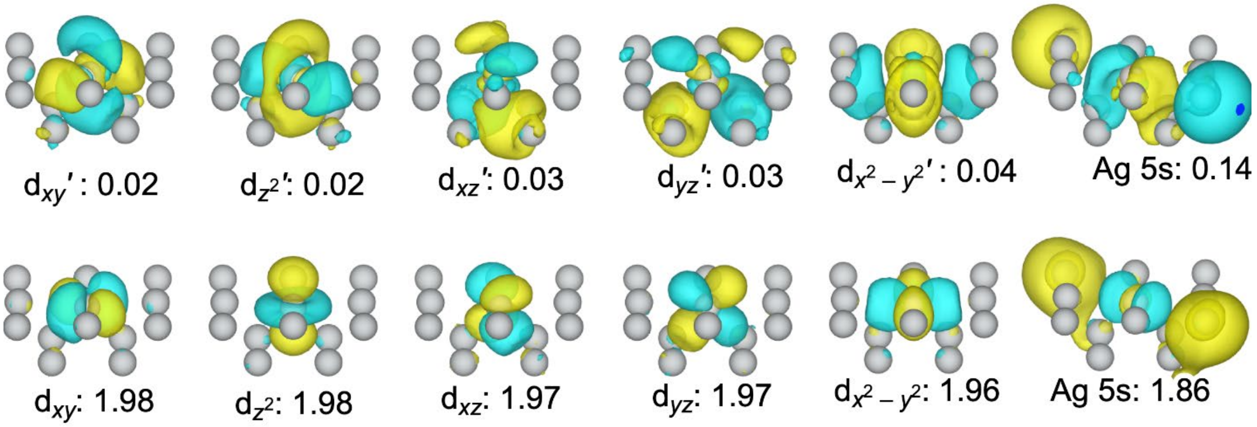

Figure S9. Emb-CASSCF(12e,12o) natural orbitals for Pt_1Ag_{12} calculated with an aug-cc-pVDZ basis set and incorporating the Ag 5s pair. The orbitals are arranged in order of decreasing occupation on the bottom row and correlating orbitals are shown in vertical pairs. The labels below each orbital isosurface give both the orbital assignment and occupation number. All the d-orbitals are centered on the Pt. The orbitals are plotted at a 0.03 Å^{-3} isosurface level.

With the AVTZ basis set, the (12e,12o) active space that incorporates the Ag 5s orbitals sees the Pt 5d orbitals all having occupation numbers of 1.99 or 0.01 (Figure S11)

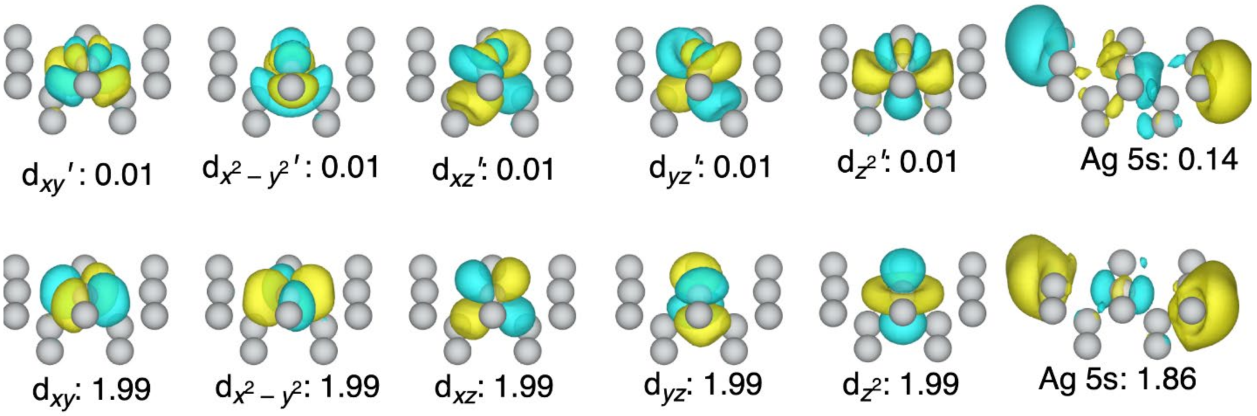

Figure S10. Emb-CASSCF(12e,12o) natural orbitals for Pt_1Ag_{12} calculated with an aug-cc-pVTZ basis set and incorporating the Ag 5s pair. The orbitals are arranged in order of decreasing occupation on the bottom row and correlating orbitals are shown in vertical pairs. The labels below each orbital isosurface give both the orbital assignment and occupation number. All the d-orbitals are centered on the Pt. The orbitals are plotted at a 0.03 Å^{-3} isosurface level.

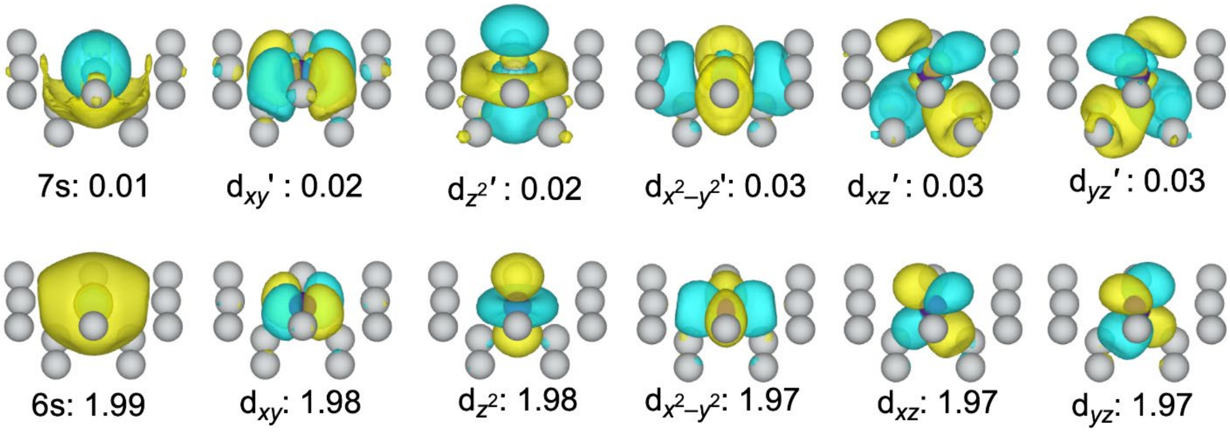

Figure S11. Emb-CASSCF(12e,12o) natural orbitals for Pt_1Ag_{12} calculated with an aug-cc-pVDZ basis set and incorporating the Pt 6s/7s pair. The orbitals are arranged in order of decreasing occupation on the bottom row and correlating orbitals are shown in vertical pairs. The labels below each orbital isosurface give both the orbital assignment and occupation number. All the d-orbitals are centered on the Pt. The orbitals are plotted at a 0.03 Å^{-3} isosurface level.

The Pt_1Ag_{12} cluster can only contribute (6e,6o) for the minimal active space of (12e,12o). The lowest energy active orbitals for the Pt_1Ag_{12} cluster when CO is desorbed are shown in Figures S12 and S13.

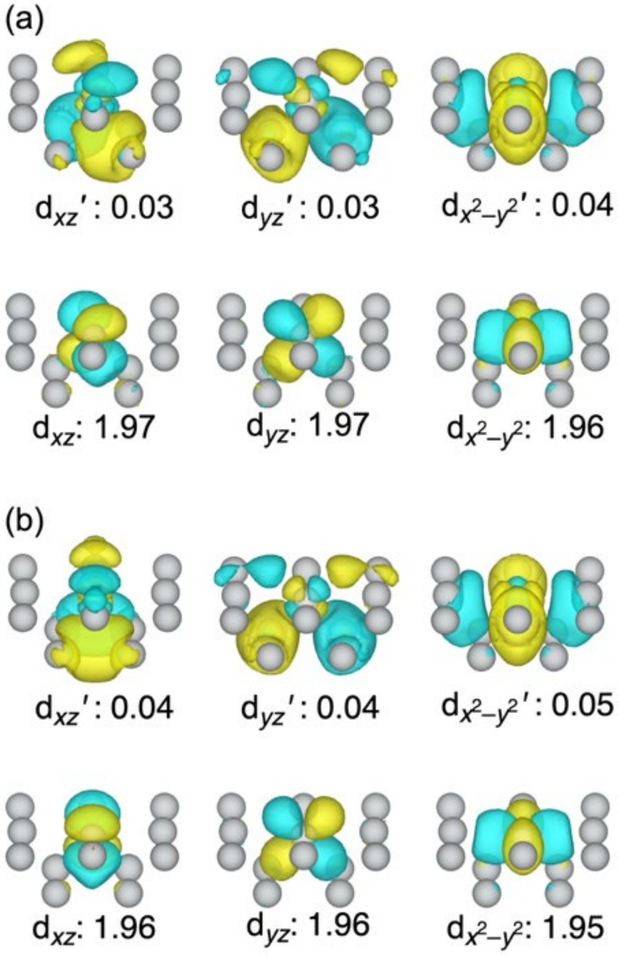

Figure S12. Emb-CASSCF(6e,6o) natural orbitals for Pt_1Ag_{12} calculated with the (a) aug-cc-pVDZ basis set and (b) with the aug-cc-pVTZ basis set. The orbitals are arranged in order of decreasing occupation on the bottom row and correlating orbitals are shown in vertical pairs. The labels below each orbital isosurface give both the orbital assignment and occupation number. All the d-orbitals are centered on the Pt. The orbitals are plotted at a 0.03 $Å^{-3}$ isosurface level.

For Pt, the largest active space is (22e,20o) and that active space incorporates the CO 3σ, 4σ, 5σ, 6σ, 1π, and 2π orbitals, all the Pt 5d orbitals, and a pair of Ag 5s orbitals. The dopant Pt–CO π/π* orbitals were assigned based both on nodal symmetry and through the analysis of natural orbital coefficients.

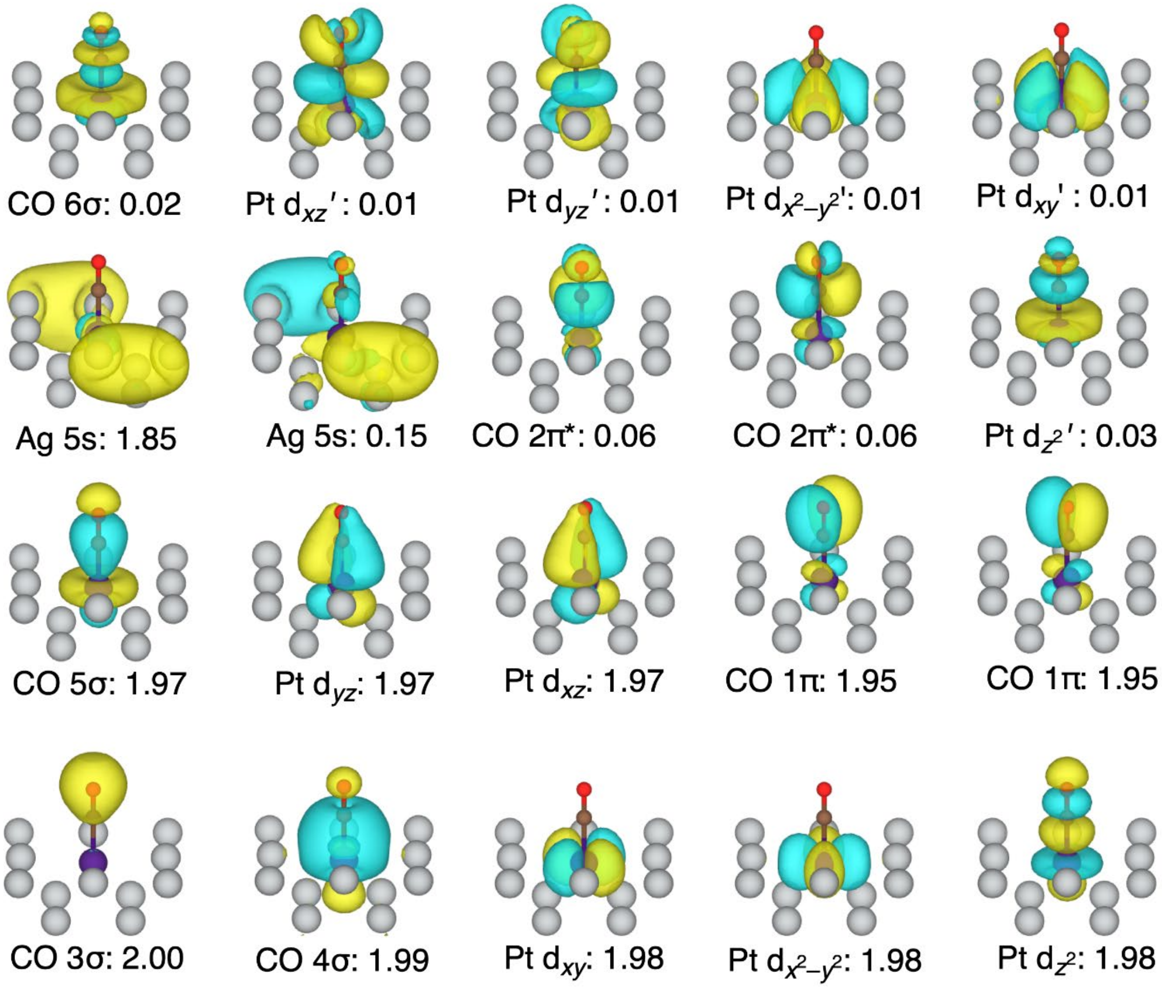

Figure S13. Emb-DMRGSCF(22e,20o) natural orbitals for CO adsorbed on Pt_1Ag_{12} calculated with an aug-cc-pVDZ basis set and a maximum bond dimension of 2000. The orbitals are arranged in order of decreasing occupation number from left to right, starting from the bottom row. The labels below each orbital isosurface give both the orbital assignment and occupation number. The orbitals are plotted at a 0.03 $Å^{-3}$ isosurface level.

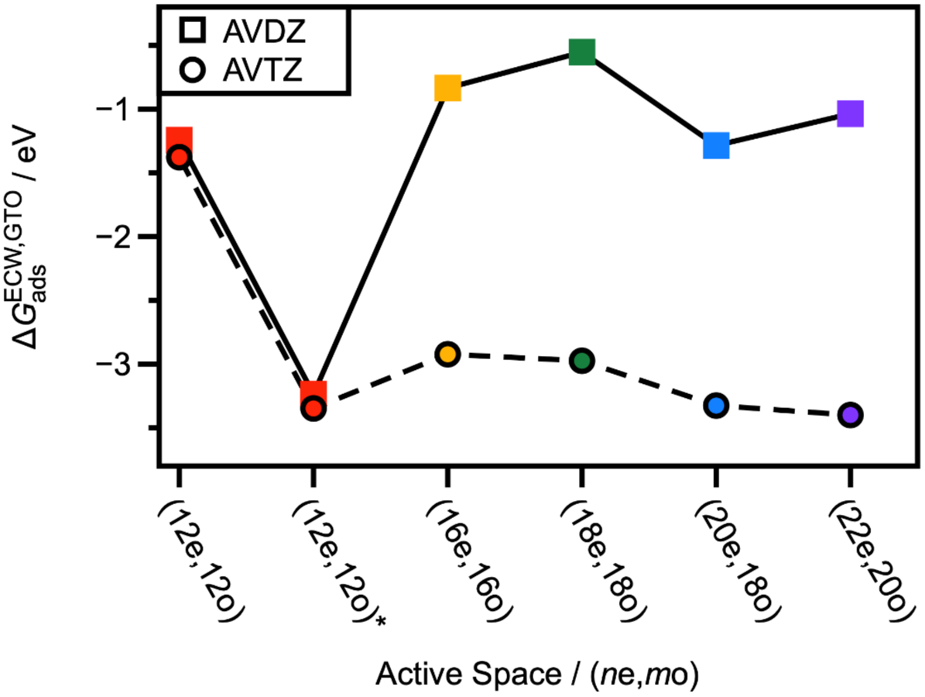

Figure S14. Adsorption free energy for CO on Pt_1Ag_{12} as a function of active space size and basis set size using a GTO basis set for the emb-DFT term in the ECW expression. Compare to Figure 5a of the main text.

Additional Data for CO on Ni_1Ag_{12}

Here, we show the optimized natural orbitals for the embedded Ni_1Ag_{12} cluster in the absence of any CO. Note, that when the desorbed CO is present in a supermolecule approach, the active spaces grow to (22e,20o), but the metal cluster's orbitals are largely unaffected. We show this active space to show how Ni 4s orbitals enter the active spaces once CO is desorbed.

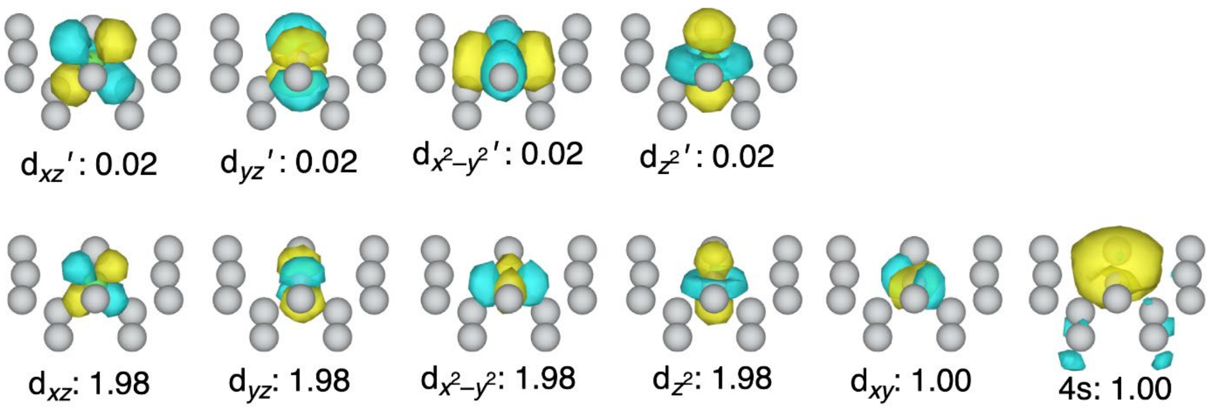

Figure S15. Emb-CASSCF(12e,12o) natural orbitals for Ni_1Ag_{12} calculated with an aug-cc-pVDZ basis set. The orbitals are arranged in order of decreasing occupation on the bottom row and correlating orbitals are shown in vertical pairs. The labels below each orbital isosurface give both the orbital assignment and occupation number. All the d-orbitals are centered on the Ni as is the 4s-orbital. The orbitals are plotted at a 0.03 Å^{-3} isosurface level.

For Ni, the largest active space is (22e,20o) and that active space incorporates the CO 3σ, 4σ, 5σ, 6σ, 1π, and 2π orbitals, all the Ni 3d orbitals, and a pair of Ag 5s orbitals. The dopant Ni–CO π/π* orbitals were assigned based both on nodal symmetry and through the analysis of natural orbital coefficients.

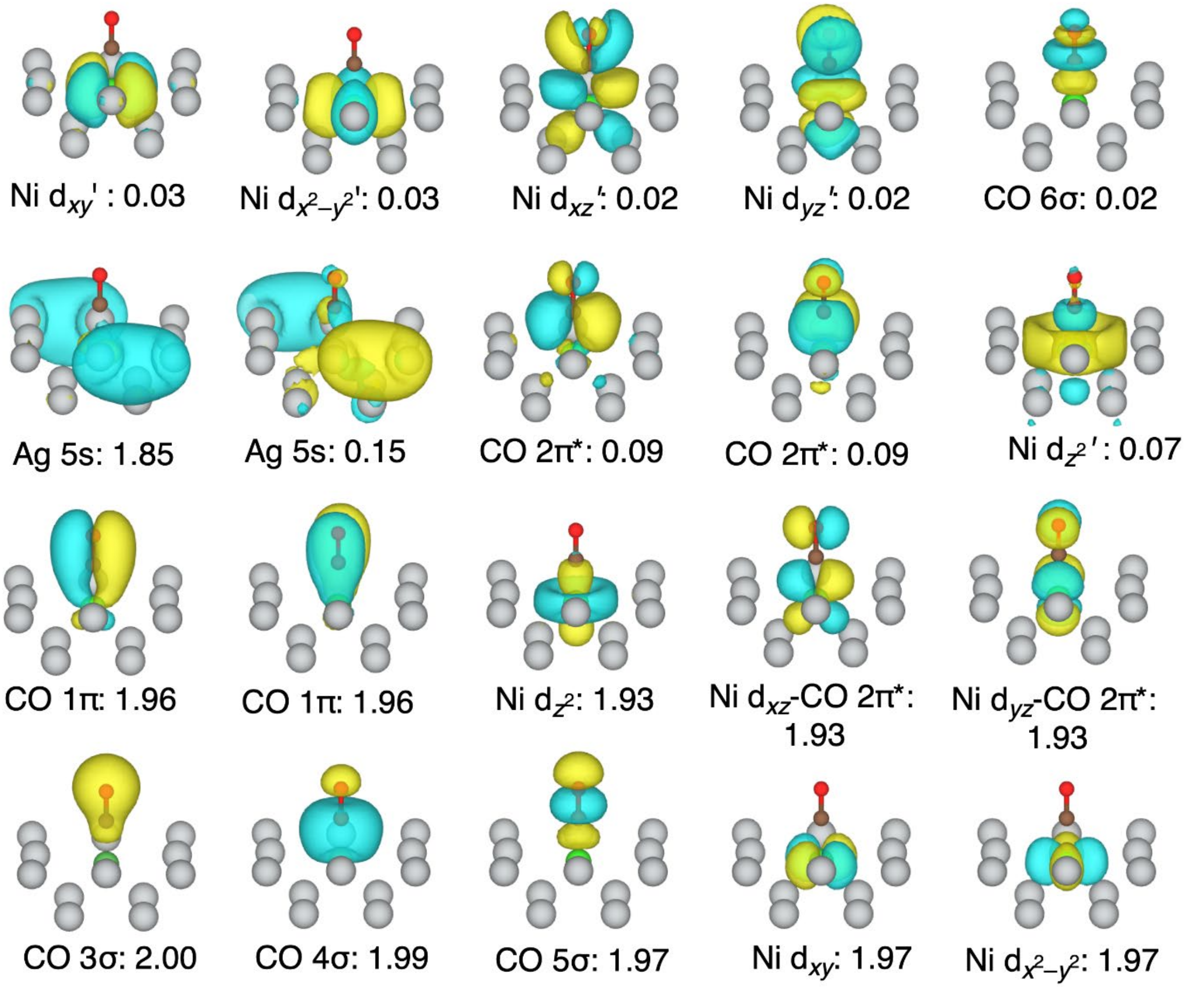

Figure S16. Emb-DMRGSCF(22e,20o) natural orbitals for CO adsorbed on Ni_1Ag_{12} calculated with an aug-cc-pVDZ basis set and a maximum bond dimension of 2000. The orbitals are arranged in order of decreasing occupation number from left to right, starting from the bottom row. The labels below each orbital isosurface give both the orbital assignment and occupation number. The orbitals are plotted at a 0.03 Å^{-3} isosurface level.

Ni, behaves differently than the other systems, as using the PW basis for the embedded DFT term leads to overly positive adsorption free energies (Figure S11). Using the GTO basis for the embedded DFT, however, leads to more reasonable adsorption free energies (see Figure 5b of the main text).

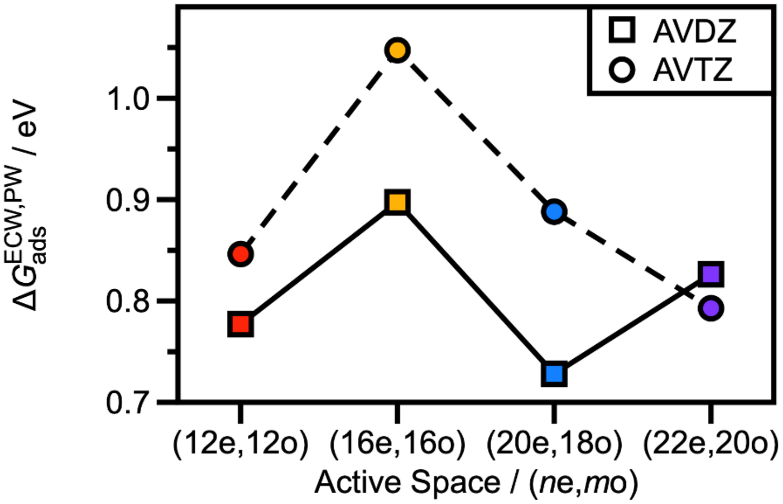

Figure S17. Adsorption free energy for CO on Ni_1Ag_{12} as a function of active space size and basis set size using a planewave (PW) basis set for the emb-DFT term in the ECW expression. Compare to Figure 5b of the main text.

References

(1) Tagliacozzo, L.; Evenbly, G.; Vidal, G. Simulation of two-dimensional quantum systems using a tree tensor network that exploits the entropic area law. *Phys. Rev. B* **2009,** *80*, 235127.
(2) Olivares-Amaya, R.; Hu, W.; Nakatani, N.; Sharma, S.; Yang, J.; Chan, G. K.-L. The ab-initio density matrix renormalization group in practice. *J. Chem. Phys.* **2015,** *142*, 034102.
